# Effects of age on psychophysical measures of auditory temporal processing and speech reception at low and high levels

**DOI:** 10.1016/j.heares.2020.108117

**Published:** 2021-02

**Authors:** Samuele Carcagno, Christopher J. Plack

**Affiliations:** a Department of Psychology, Lancaster University, Lancaster, LA1 4YF, United Kingdom; b Manchester Centre for Audiology and Deafness, University of Manchester, Academic Health Science Centre, M13 9PL, United Kingdom

**Keywords:** Cochlear synaptopathy, Presbycusis, Temporal coding, Hearing loss, Speech reception, Pitch

## Abstract

Age-related cochlear synaptopathy (CS) has been shown to occur in rodents with minimal noise exposure, and has been hypothesized to play a crucial role in age-related hearing declines in humans. It is not known to what extent age-related CS occurs in humans, and how it affects the coding of supra-threshold sounds and speech in noise. Because in rodents CS affects mainly low- and medium-spontaneous rate (L/M-SR) auditory-nerve fibers with rate-level functions covering medium-high levels, it should lead to greater deficits in the processing of sounds at high than at low stimulus levels. In this cross-sectional study the performance of 102 listeners across the age range (34 young, 34 middle-aged, 34 older) was assessed in a set of psychophysical temporal processing and speech reception in noise tests at both low, and high stimulus levels. Mixed-effect multiple regression models were used to estimate the effects of age while partialing out effects of audiometric thresholds, lifetime noise exposure, cognitive abilities (assessed with additional tests), and musical experience. Age was independently associated with performance deficits on several tests. However, only for one out of 13 tests were age effects credibly larger at the high compared to the low stimulus level. Overall these results do not provide much evidence that age-related CS, to the extent to which it may occur in humans according to the rodent model of greater L/M-SR synaptic loss, has substantial effects on psychophysical measures of auditory temporal processing or on speech reception in noise.

## Highlights

• We found little evidence of greater age-related hearing declines at high sound levels.
• There are age-related temporal-processing declines independent of hearing loss.
• No evidence of age-related speech-reception deficits independent of hearing loss.

## 1 Introduction

Presbycusis, the decline of hearing abilities with age, is one of the most common chronic conditions in older adults (Lin et al., 2011). The hearing difficulties of older adults can be in part explained by their reduced sensitivity to low-level sounds, which is usually due to cochlear hair cell dysfunction caused by age-related metabolic changes in the cochlea, cumulative effects of noise exposure, ototoxic agents, or other factors (Schmiedt, 2010). However, presbycusis is also associated with supra-threshold deficits that cannot be easily explained by hair cell dysfunction (Fitzgibbons, Gordon-Salant, 2010, Humes, Dubno, 2010). Age-related cochlear synaptopathy (CS), a condition affecting the synapses between inner hair cells (IHCs), and auditory-nerve fibers, has been hypothesized to play a major role in these supra-threshold deficits (Kujawa, Liberman, 2015, Liberman, Kujawa, 2017).

CS and its phenomenology have been primarily characterized in rodents. Noise exposures titrated to cause only temporary threshold shifts, with little outer hair cell (OHC) damage, have been shown to result in a permanent loss of synapses between IHCs and auditory-nerve fibers in young CBA/CaJ mice (Kujawa and Liberman, 2009). This synaptic disconnection has been shown to affect mostly auditory-nerve fibers with low and medium spontaneous rates (L/M-SR fibers) with rate-level functions extending to high levels, beyond the saturation point of high spontaneous rate (H-SR) fibers (Furman et al., 2013). Noise-induced CS has been observed in a range of rodent species other than CBA/CaJ mice (see Hickox et al., 2017, for a review), and in primates (Valero et al., 2017).

CS has also been observed in aged CBA/CaJ mice raised in a quiet environment before the onset of OHC loss (Sergeyenko et al., 2013). Besides CBA/CaJ mice (Parthasarathy, Kujawa, 2018, Sergeyenko, Lall, Liberman, Kujawa, 2013), age-related CS has been observed in gerbils (Gleich et al., 2016), and it has been shown to interact with noise exposure, with noise exposures at a young age leading to accelerated age-related CS (Fernandez et al., 2015).

Evidence consistent with age-related CS in humans comes from post-mortem studies of temporal bones. After synaptic disconnection, the peripheral axons of spiral ganglion neurons (SGNs) degenerate, followed later by a loss of SGN bodies. Post-mortem studies of human temporal bones have shown steady age-related declines of SGN peripheral axons (Wu et al., 2019), IHC synaptic ribbons (Viana et al., 2015), and SGN bodies (Makary et al., 2011), that precede or exceed hair cell loss. It is not known whether the age-related degeneration of SGNs found in human temporal bones mainly affects L/M-SR fibers.

Several pieces of evidence suggest that age-related CS in rodents may be selective for L/M-SR fibers. Schmiedt et al. (1996) found a drastic decline in the proportion of L-SR fibers in quiet-aged gerbils, although this decline occurred only for fibers with characteristic frequencies >6 kHz. Stamataki et al. (2006) found a preferential age-related loss of small afferent terminals (that correspond to L-SR fibers) on IHCs in a mouse strain which exhibits a pattern of progressive age-related hearing loss similar to that of human presbycusis (C57BL/6J mice; Fetoni, Picciotti, Paludetti, Troiani, 2011, Someya, Xu, Kondo, Ding, Salvi, Yamasoba, Rabinovitch, Weindruch, Leeuwenburgh, Tanokura, Prolla, 2009). Shrestha et al. (2018) used single-cell ribonucleic acid sequencing to characterize SGNs, and derived three subtypes of SGNs on the basis of this molecular characterization: types Ia, Ib, and Ic, that respectively match the anatomical features of H-, M-, and L-SR SGNs. Molecular characterization at three different ages (32, 64, and 128 weeks) revealed that the type Ic neurons (matching the L-SR profile) are selectively vulnerable to age-related loss.

Phenomenologically the picture regarding L/M-SR specificity in age-related CS is less clear. Although ABR wave I growth functions with click level (either in dB SPL or dB sensation level) always appear markedly shallower in mice with age-related CS on a linear scale, on a log scale they sometimes appear to have a similar shape to those of young mice, with a downward offset at all levels (Möhrle, Ni, Varakina, Bing, Lee, Zimmermann, Knipper, Rüttiger, 2016, Parthasarathy, Kujawa, 2018, Sergeyenko, Lall, Liberman, Kujawa, 2013). However, the age-related wave I declines at low stimulus levels may reflect, at least in part, residual OHC dysfunction (which would not greatly affect wave I responses at high stimulus levels; Cheatham et al., 2004, Chen, Tanaka, Henderson, 2008, Verhulst, Jagadeesh, Mauermann, Ernst, 2016, Verhulst, Altoe, Vasilkov, 2018). Similarly, frequency following response growth functions with level appear to have a similar shape for mice with age-related CS and young controls, with a downward offset for aged mice at all levels (Parthasarathy and Kujawa, 2018). However, if the likely effect of OHC dysfunction is compensated for by plotting the functions at equal sensation levels, the functions appear markedly shallower in aged animals compared to young controls (Parthasarathy and Kujawa, 2018).

Unlike deficits due to OHC dysfunction, which should affect mainly the processing of sounds at low stimulus levels, a predominant involvement of L/M-SR fibers in CS should cause deficits mainly at high stimulus levels according to several theoretical models (Bharadwaj, Verhulst, Shaheen, Liberman, Shinn-Cunningham, 2014, Plack, Leger, Prendergast, Kluk, Guest, Munro, 2016). These models hypothesize a direct role of L/M-SR fibers in the coding of high-level stimuli. Although another theoretical model, proposed by Carney (2018), excludes a direct involvement of L/M-SR fibers in the coding of high-level stimuli, it envisages an important role for these fibers in a feedback loop that is crucial for the optimal encoding of high-level stimuli. Thus, also for this model it is reasonable to expect that deafferentiation of L/M-SR fibers should lead to deficits occurring mainly at high stimulus levels.

As mentioned before, there is evidence of age-related supra-threshold auditory deficits in humans, but while CS is certainly a candidate explanation of these deficits, they may be also due to age-related changes of the central auditory system (Caspary, Ling, Turner, Hughes, 2008, Ouda, Profant, Syka, 2015), or to age-related cognitive declines (Dryden, Allen, Henshaw, Heinrich, 2017, Humes, Dubno, 2010, Kamerer, AuBuchon, Fultz, Kopun, Neely, Rasetshwane, 2019, Schneider, Pichora-Fuller, Daneman, 2010). Studies showing greater age-related declines at high compared to low stimulus levels would provide stronger evidence of age-related CS in humans, if it is assumed that age-related CS in humans affects mainly L/M-SR fibers. In line with previous studies of age-related CS in humans (Garrett, Verhulst, 2019, Grose, Buss, Elmore, 2019, Johannesen, Buzo, Lopez-Poveda, 2019, Prendergast, Couth, Millman, Guest, Kluk, Munro, Plack, 2019), this will be the working assumption on which the evidence for age-related CS in the current study will be assessed. However, it should be kept in mind that, although the evidence reviewed above provides substantial support for this assumption, this evidence is not conclusive.

Only a few studies have assessed the differential effects of age on auditory processing at high and low stimulus levels. Grose et al. (2019) compared, between groups of young and older listeners (∼10 per group), thresholds for sinusoidal amplitude modulation (AM) detection with a 2-kHz tonal carrier in quiet and in noise, and thresholds for spectral modulation detection with a two-octave noise band, at two levels (70 and 85 dB SPL for AM detection; 65 and 85 dB SPL for spectral modulation detection). They did not find either significant effects of age or significant age by level interactions for either task.

Moore and Vinay (2019) compared thresholds for envelope regularity discrimination between 10 young and 10 older listeners at levels of 20 dB SL, and 80 dB SPL. While they found significant effects of age, with raised thresholds in older listeners, the effect of age was not significantly larger at the high stimulus level.

Prendergast et al. (2019) tested 156 young and middle-aged (<60 years old) participants on two psychophysical tasks (sinusoidal AM detection with a 4-kHz tonal carrier, and interaural phase difference detection for the envelope of a transposed tone with a 4-kHz carrier and a 255-Hz modulator), and two speech-reception tasks [coordinate response measure (CRM; Bolia et al., 2000) with two diotic speech maskers, and digit triplets test (DTT; Smits et al., 2004) with a speech-shaped noise masker] at both low and high sound levels. They found that multiple regression models including age, lifetime noise exposure, and 16-kHz audiometric thresholds significantly predicted differential performance at the high vs low stimulus levels in the AM detection and DTT tasks. The relation between age and differential thresholds, however, was opposite in the two tasks. While for the DTT increasing age was associated with higher relative thresholds at the high stimulus level, for the AM detection task increasing age was associated with lower relative thresholds at the high stimulus level. Subsequent analyses suggested that these effects remained stable when audiometric thresholds at the frequency that best correlated with the differential measure (4 kHz for AM detection, and 0.5 kHz for DTT) were used as a covariate instead of 16-kHz audiometric thresholds.

Johannesen et al. (2019) measured speech reception thresholds (SRTs) in noise for sentences and for words in a group of 94 participants ranging in age from 12 to 68 years. SRTs were measured for both steady-state, and fluctuating noise maskers with the target speech fixed at a level of 65 dB SPL in the case of sentence SRTs, and at levels of 50, 65 or 75 dB SPL in the case of word SRTs. Sentence SRTs were not significantly correlated with age. Word SRTs significantly worsened with increasing age, but there was no evidence of larger age effects at higher target speech levels (the effect of age did not interact significantly with target speech level). Furthermore, SRTs were not significantly correlated with the slope of ABR wave I amplitude growth with level — a putative electrophysiological measure of CS (Bramhall, Beach, Epp, Le Prell, Lopez-Poveda, Plack, Schaette, Verhulst, Canlon, 2019).

Overall, the studies that have compared the effects of age on auditory processing at high and low stimulus levels have yielded mixed findings. Two of these studies (Grose, Buss, Elmore, 2019, Moore, Vinay, 2019) did not find evidence of differential effects of age on auditory processing at high and low stimulus levels, but their sample sizes were small. The Prendergast et al. (2019) study had a much larger sample size, but did not include participants over the age of 60, and found differential effects of age at high and low stimulus levels consistent with age-related CS in only one of four tasks (and a differential effect in the direction opposite to that predicted by age-related CS in another task). The differential effect of age consistent with CS in the Prendergast et al. (2019) was found on a speech reception in noise task, but the Johannesen et al. (2019) study, that also had a large sample size and included participants over a wide age range, did not find differential effects of age at high and low stimulus levels consistent with age-related CS on speech reception in noise tasks.

The primary aim of the current study was to test the hypothesis that age-related deficits in the processing of supra-threshold sounds are greater in conditions that are thought to rely on L/M-SR fibers for stimulus coding: namely stimuli presented at a high SPL, and within a noise background. To address this aim we assessed the performance of 102 listeners across the age range on a battery of psychophysical and speech-reception tests, with each test run at a low, and at a high stimulus level.

CS is expected to degrade the precise timing information (Bharadwaj, Verhulst, Shaheen, Liberman, Shinn-Cunningham, 2014, Lopez-Poveda, Barrios, 2013, Plack, Barker, Prendergast, 2014) that is often crucial for the encoding of basic sound attributes, such as the pitch of low-frequency tones, and the spatial location of tones with interaural time differences (Moore, 2008, Moore, 2019). For this reason the psychophysical test battery of the current study focused on tasks that are thought to require precise temporal coding: AM detection for tones with unresolved side bands, frequency discrimination for low-frequency pure tones, fundamental frequency (F0) discrimination for unresolved complex tones, and interaural phase difference (IPD) detection.

Older people often complain of difficulties understanding speech in noise, and age-related CS has been hypothesized to play a major role in these difficulties (Kujawa, Liberman, 2015, Liberman, Kujawa, 2017, Sergeyenko, Lall, Liberman, Kujawa, 2013). For this reason, the test battery of the current study also included two speech-reception tests: the DTT with a noise masker, and the CRM with two colocated or spatially offset speech maskers. The test battery also included: a test on the subjective preference for the consonance of musical intervals, a trait that has been previously associated with age-related temporal coding deficits (Bones and Plack, 2015); a questionnaire that evaluated self-reported hearing abilities; and a questionnaire estimating lifetime noise exposure.

Besides testing for age effects on differential high-low level measures, a secondary aim of this study was to assess, at each stimulus level, the contributions of age, independent of hearing sensitivity, lifetime noise exposure, cognitive abilities, and musical experience on performance in the tasks outlined above. For this reason, the test battery included also several cognitive tests, and a measure of self-reported musical experience.

The tests described in this article were part of a larger study that included electrophysiological measures of temporal coding, on the same cohort of participants. This paper will present only the results of the behavioral tests. The results of the electrophysiological tests have been published previously (Carcagno and Plack, 2020). Those results did not provide evidence that age-related declines in electrophysiological responses, restricted to a low-frequency test region (≲ 3 kHz) with near-normal audiometric thresholds across the age range, were larger at high compared to low stimulus levels. Thus, assuming that age-related CS affects mainly L/M-SR fibers, those results do not provide evidence of age-related CS occurring within the low-frequency test region. However, this does not warrant the stronger conclusion that age-related CS was not occurring in this frequency region, because the sensitivity of electrophysiological measures to CS in humans is uncertain (Bramhall, Beach, Epp, Le Prell, Lopez-Poveda, Plack, Schaette, Verhulst, Canlon, 2019). The behavioral and speech-reception tests reported in the current paper provide a further, independent test of the hypothesis that responses to supra-threshold sounds in humans match a profile consistent with the predictions of age-related CS specific for L/M-SR fibers.

## 2 Methods

### 2.1 Participants

A total of 170 participants from three age groups (young: 18–39, middle-aged: 40–59, older: >60 years old) were enrolled in the study. Sixty-eight participants either failed to meet the selection criteria outlined below, or withdrew from the study. Only the data of the 102 participants who completed the study will be presented. Selection criteria included audiometric thresholds for both ears below 20 dB HL at octave frequencies from 0.125 to 2 kHz, and below 40 dB HL at 4 kHz. No selection criteria were imposed for frequencies above 4 kHz. Due to the use of an incorrect calibration table for the headphones used in the audiometric tests the actual cutoff thresholds differed by a few dBs with respect to the nominal cutoff thresholds listed above. Using the correct calibration table five older, two middle-aged, and two young participants would not have passed the selection. However, these listeners had thresholds below 30.5 dB HL for audiometric frequencies up to 2 kHz, and below 37 dB HL at 4 kHz. Given that their thresholds were only slightly above the cutoff criteria, and given that audiometric thresholds were used as continuous covariates, the data of these listeners were included in the analyses. Participants with audiometric threshold asymmetries between the left and right ear larger than 20 dB at any frequency from 0.125 to 4 kHz were excluded from the study. Overall the selection criteria ensured that all participants had near-normal hearing in a low-frequency region extending up to 2 kHz. An otoscopic examination was performed prior to the beginning of the tests, and participants with earwax occlusions were excluded from the study. Participants were required to be native British English speakers.

Recruitment continued until 34 participants from each age group had completed the study. Within each age group 27 females, and seven males completed the study. Towards the end of the study, recruitment was targeted to ensure that the proportion of females to males would be the same across the three age groups. We are not aware of interactions between age-related CS and sex that could limit the applicability of the findings from the current study, in which a greater proportion of females than males was tested, to the general population. The youngest participant was 18.8, while the oldest was 73.6 years old.

Participants were asked to report the number of years of musical practice (with a musical instrument or vocal) they had. They gave written informed consent for participation in the study, and received an hourly wage. All the experimental procedures were approved by the Lancaster University Research Ethics Committee.

### 2.2 Noise exposure

Lifetime noise exposure was estimated via the structured interview developed by Lutman et al. (2008), which estimates the duration and level of noise exposure for a range of activities. One unit of noise exposure calculated via the interview corresponds to an eight hour daily exposure, for five days a week, for 52 weeks, for a year, to a noise level of 90 dBA. The estimated noise exposure was summed across all activities (occupational or recreational) to estimate the total cumulative noise exposure (TCNE). For the analyses the TCNE was log-transformed using base 10, so that a unit difference in the log_10_-transformed TCNE corresponds to a tenfold difference in noise exposure energy. Further details of the noise exposure interview are available in a previous publication (Carcagno and Plack, 2020).

### 2.3 Audiometric thresholds

Audiometric thresholds were measured for pure tones at octave frequencies from 0.125 to 8 kHz (clinical frequency range) as well as for pure tones at 12 and 16 kHz (extended high-frequency range) using a two-interval two-alternative forced-choice (2I-2AFC) task with an adaptive two-down one-up transformed up-down procedure tracking the 70.7% correct point on the psychometric function (Levitt, 1971). Further details of the procedure are available in a previous publication (Carcagno and Plack, 2020).

### 2.4 Psychophysical and speech tasks

Psychophysical and speech reception tests were run with n-interval m-alternative forced-choice tasks using the updated maximum likelihood (UML) adaptive procedure (Shen and Richards, 2012). The UML is a Bayesian procedure that starts with experimenter-defined priors of the free parameters of the psychometric function (threshold, slope, and lapse rate; the guess rate for forced-choice tasks is fixed by the number of response alternatives); the priors chosen for each task are listed in the supplementary materials. The procedure updates the posterior distribution of the psychometric function after each response. On each trial the stimulus is placed at one of the four sweetpoints that allow the most efficient sampling for these parameters. For the current study, a 2-down 1-up sweetpoint selection rule was used. After data collection, listeners’ responses were re-fit (as suggested in Shen, Dai, Richards, 2015, Shen, Richards, 2012) through Markov Chain Monte Carlo (MCMC) methods to estimate their psychometric functions (Kuss et al., 2005). A significant advantage of this approach based on the UML procedure and threshold estimation through psychometric function fits is that the fits take into account the lapse rate, which could otherwise bias threshold estimates (Wichmann and Hill, 2001).

For the frequency/F0 discrimination and the IPD detection tasks stimuli were presented with the AAAA vs ABAB paradigm of Hopkins and Moore (2010), which has been shown to minimize practice effects (King et al., 2013). In this paradigm the standard interval(s) contains four identical stimuli (AAAA), while the comparison interval contains stimuli alternating along the dimension of interest (ABAB). The silent interval between each of the four stimuli was 20 ms. The silence between observation intervals in these tasks, as well as in the AM detection task, had a duration of 500 ms.

Stimuli for the psychophysical tasks had a frequency (or were centered at a frequency) of either 0.6 kHz (low-frequency stimuli), or ∼ 2 kHz (high-frequency stimuli). Two bands of pink noise were added to the stimuli. One band was lowpass filtered at a frequency ∼25% below the frequency of the stimulus (450 Hz for the low-, and 1500 Hz for the high-frequency stimuli, respectively), with a spectrum level of 45 dB SPL at 100 Hz for the high-level stimuli, and a spectrum level of 5 dB SPL at 100 Hz for the low-level stimuli. The second band was bandpass filtered between 3 and 8 kHz, and had a spectrum level of 40 dB SPL at 4 kHz for the high-level stimuli, and a spectrum level of 0 dB SPL at 4 kHz for the low-level stimuli. The high-frequency band served to limit upward spread of excitation, thus reducing the contribution of off-frequency H-SR fibers in the coding of the stimuli. The low-frequency band served to mask the low-frequency combination tones generated by stimuli with multiple frequency components (complex tones and amplitude-modulated tones).

Participants first completed one block of 25 practice trials for each condition of the psychophysical tasks to familiarize themselves with the stimuli and the tasks; the data from these practice trials were excluded from the analyses. This familiarization phase was typically run during their first session. No practice blocks were run for the speech reception tasks, but the experimenter made sure that participants had understood the instructions by running a few trials with them immediately prior the beginning of each speech test. For both the psychophysical and the speech tests participants completed two blocks of 80 trials for each stimulus condition over a number of sessions. At the end of each trial, feedback was provided by means of a colored light (green=correct, red=incorrect) on the computer screen.

#### 2.4.1 Amplitude modulation detection

AM detection was assessed with a 3I-3AFC task for 2-kHz carriers sinusoidally modulated at rates of 25, 50, or 100 Hz. The amplitude-modulated tones had a duration of 320 ms, including 10-ms onset and offset cosine-squared ramps. The unmodulated carriers had levels of 40, or 80 dB SPL^1^. The starting and maximum possible difference in AM depth was 100%. The stimuli were presented diotically.

#### 2.4.2 Frequency/F0 discrimination

Frequency and F0 discrimination were measured with a 2I-2AFC task using the AAAA vs ABAB paradigm (with B tones having a higher frequency/F0 than A tones). Frequency discrimination was measured for 0.6, and 2 kHz pure tones, at levels of 40, and 80 dB SPL. The tones were presented diotically and had a duration of 300 ms, including 10-ms onset and offset cosine-squared ramps.

F0 discrimination was measured for complex tones with an F0 of 100 Hz, and harmonics added in sine phase. The tones were bandpass filtered between 1.5 and 2.5 kHz, and had an overall level of 40, or 80 dB SPL.

The frequency of the B pure tones ($f_{B}$) was initially set 10% higher than the frequency of the A pure tones ($f_{A}$) [i.e. $f_{B}=f_{A}+f_{A}\cdot 10/100$]. The F0 of the B complex tones ($F0_{B}$) was initially set 80% higher than the F0 of the A complex tones ($F0_{A}$) [i.e. $F0_{B}=F0_{A}+F0_{A}\cdot 80/100$]. The maximum permitted frequency/F0 difference was in each case 99%.

The rank of the lowest harmonic was 15 for the A complex tones. The lowest harmonic rank of the B complex tones decreased as a function of their F0, but was never lower than 8. The complex tones thus contained exclusively, or mainly (for trials with very large F0 differences), unresolved harmonics (Moore and Gockel, 2011).

#### 2.4.3 Interaural phase difference detection

IPD detection was assessed with a 2I-2AFC task using the AAAA vs ABAB paradigm (with A tones having an IPD of 0° and B tones having an IPD >0°). IPD detection was assessed by introducing an IPD to the modulator (MOD) of 100% sinusoidally amplitude-modulated tones at carrier frequencies of 0.6 and 2 kHz, with a modulation rate of 100 Hz. IPD detection was also assessed in separate conditions in which an IPD was applied to 0.6-kHz pure tones. The conditions in which the IPD was applied to the modulator of an AM tone will be referred to as MOD, while the conditions in which the IPD was applied to pure tones will be referred as PT. In the 2-kHz MOD conditions the IPD should be detectable only in the envelope (ENV) of the stimuli because the sidebands are not spectrally resolved and their frequency would in any case be too high (Brughera et al., 2013) for the detection of IPDs in the temporal fine structure (TFS). In the 0.6-kHz PT conditions, on the other hand, only IPDs in the TFS are available. In the 0.6-kHz MOD conditions, because the sidebands may be spectrally resolved and are of relatively low frequency, IPDs in both the ENV and the TFS could be available to the listener to perform the task. The tones for both the MOD and PT conditions had a duration of 400 ms, including 50 ms onset and offset cosine-squared ramps, and were presented at levels of 40, or 80 dB SPL. The initial and maximum possible IPD was 180°.

#### 2.4.4 Coordinate response measure

On each trial participants were presented with three (one target, two interfering) simultaneous sentences from a version of the CRM corpus (Bolia et al., 2000) recorded by eight (four males and four females) native British English speakers (Kitterick et al., 2010). The sentences were of the form “Ready < call sign > go to < color > < number > now”. There were eight possible call signs (“arrow”, “baron”, “charlie”, “eagle”, “hopper”, “laker”, “ringo”, “tiger”), four possible colors (blue, red, white, green), and four possible numbers (1–4). The three speakers were drawn randomly from the set of eight available speakers on each trial (hence the identity of the target speaker changed across trials). The sentence spoken by the speaker using the call sign “baron” was defined as the target sentence (the interfering speakers used different call signs randomly drawn from the set of the seven remaining call signs). Participants were presented with a 4x4 matrix of colored and numbered buttons corresponding to each possible color/number combination, and were asked to click on the button corresponding to the color/number combination specified in the target sentence. The colors and numbers spoken by the interfering speakers were randomly drawn from the available set of colors and numbers, and could occasionally match the color and/or number in the target sentence.

The sentences were spatialized by convolving them with the head-related impulse responses of subject #3 from the CIPIC database (Algazi et al., 2002). The target sentence was always presented at a 0° azimuth. In the colocated condition the interfering sentences were also presented at a 0° azimuth. In the offset condition one of the interfering sentences was presented at a +65°, and the other at a -65° azimuth. In the high-level condition each interfering sentence had a root mean square (RMS) level of 74 dB SPL, while in the low-level condition each had an RMS level of 39 dB SPL.

The sentences were lowpass filtered at 3 kHz, and three bands of pink noise were added to the sentences to eliminate the contribution of high-frequency cochlear regions to the coding of the sentences. The three noise bands were all bandpass filtered between 3 to 8 kHz, but each had the same azimuth as one of the sentences (all a 0° azimuth in the colocated conditions; -65°, 0°, and +65° azimuth in the offset conditions). The noise bands had each a level of 40 dB SPL at 4 kHz in the high-level conditions, and a level of 5 dB SPL at 4 kHz in the low-level conditions.

The target sentence was initially presented at a signal to noise ratio (SNR) of 7 dB (with the noise level defined as the combined level of the two masker sentences), and its level was varied adaptively to define the psychometric function.

#### 2.4.5 Digit triplets test

On each trial the participant was presented with three digits in the 1–9 range, but excluding 7 (the only digit consisting of two syllables). No repetitions of the same digit were allowed in a trial. The digits were voice recordings of a male speaker taken from McShefferty et al. (2013). The digits were lowpass filtered at 3 kHz. A noise^2^ lowpass filtered at 3 kHz, and with an RMS level of 80, or 45 dB SPL was presented throughout the duration of the trial and served as the masker. A pink noise bandpass filtered between 3 and 8 kHz was added to the stimuli to eliminate the contribution of high-frequency cochlear regions to the coding of the digits. Each trial started with the recording of a female voice saying the phrase “the digits”, and was followed by the presentation of the digits spoken by the male voice. Participants were asked to input the three digits they heard, or give their best guess if they could not hear them clearly, using a numeric keypad. Responses with repeated digits within the same sequence were not allowed.

The digits were initially presented at an SNR of 10 dB, and their level was varied adaptively to define the psychometric function.

### 2.5 Consonance preference

Participants rated the pleasantness of dyads consisting of a low (“root”) note, and a high (“interval”) note, forming a musical interval on the equal temperament (ET) scale that was either consonant [perfect fifth (P5); 700 cents)], or dissonant [tritone (TT); 600 cents]. The dyads were rated on a scale ranging from -3 to +3 in 0.1 steps by moving, via a computer mouse, a slider presented on a computer monitor (Carcagno, Lakhani, Plack, 2019, McDermott, Lehr, Oxenham, 2010). Each note consisted of an equal amplitude complex tone lowpass filtered at 2.5 kHz, with harmonics summed in sine phase. The dyads had a duration of 2 seconds, including 10-ms onset and offset cosine-squared ramps. The F0 of the root note could take one of eight possible values, from 146.83 Hz (note D3 on the ET scale) to 220 Hz (note A3 on the ET scale) in 100-cent steps. The dyads were presented diotically at a level of either 40, or 80 dB SPL. They were presented with a pink noise bandpass filtered between 3 and 8 kHz to eliminate the contribution of high-frequency cochlear places to the coding of the stimuli; the noise had a spectrum level at 4 kHz that was 40 dB below the level of the dyad. On each trial, a 2-s pink noise was presented before the dyad to weaken the sensory memory trace of the preceding dyad and minimize any effect it might have on the rating of the upcoming dyad (Bones, Hopkins, Krishnan, Plack, 2014, McDermott, Lehr, Oxenham, 2010). This noise was bandpass filtered between 0.02 and 8 kHz, and had a spectrum level at 1 kHz 40 dB below the level of the dyad. The dyad was separated from this noise by a 500-ms silent interval.

Participants completed first 16 practice trials (four for each combination of musical interval and dyad level, with F0s randomly drawn from the set of eight possible F0s). They then completed two trials for each combination of musical interval, dyad level, and F0. Both the practice and the main trials were blocked by dyad level, with the starting levels randomly chosen across participants.

The pleasantness ratings of each participant were converted to z scores by subtracting the mean and scaling by the standard deviation of the scores given by that listener across all stimulus conditions (Carcagno, Lakhani, Plack, 2019, McDermott, Lehr, Oxenham, 2010). Consonance preference was computed as the difference of these z scores between ratings of the perfect fifth and ratings of the tritone intervals.

### 2.6 Speech, spatial and qualities of hearing scale

Self-reported hearing abilities were assessed with the short version of the “Speech, Spatial and Qualities of Hearing Scale” (SSQ12; Noble et al., 2013).

### 2.7 Cognitive tests

There is evidence that cognitive abilities are associated not only with speech-reception in noise (Dryden et al., 2017), but also with performance in psychoacoustical tasks (Zhang et al., 2016). For this reason cognitive abilities were assessed with four cognitive tests, targeting mainly working memory, and fluid intelligence. Working memory has been found to be associated with speech-reception in noise in older hearing-impaired listeners (Akeroyd, 2008), and plays a prominent role in some auditory-cognitive models of speech understanding (Rönnberg et al., 2013). Fluid intelligence is a crucial aspect of general cognitive ability, and as such its assessment can provide a more rounded view of the cognitive status of an individual compared to the assessment of more specific cognitive domains (Deary, Penke, Johnson, 2010, Kovacs and Conway, 2019, Valentin Kvist and Gustafsson, 2008).

The four cognitive tests were the digit span forward and digit span backward tests (Wechsler, 1997), the reading span task (Rönnberg et al., 1989), and the Raven’s progressive matrices test (Raven and Raven, 2003). A brief description of the cognitive domains tapped by each test is given below, although it should be kept in mind that cognitive tests generally are not “pure” measures of a single cognitive construct, and that theories related to cognitive constructs are continually evolving and often there is considerable controversy regarding the cognitive processes underlying a given ability.

The digit span forward test is generally thought to measure verbal short-term memory. The digit span backward test is also dependent on verbal short-term memory. However, because it additionally requires active manipulation of the items to be recalled (transposition of their order), it is considered by some authors to be a measure of working memory (Aben et al., 2012). The reading span test is a complex span task that requires both storage and active processing of information, and as such, is generally considered a measure of working memory (Conway, Kane, Bunting, Hambrick, Wilhelm, Engle, 2005, Richardson, 2007). Performance on the reading span task has been found to be associated with speech reception in noise (Akeroyd, 2008), although this association seems to be limited to older and/or hearing-impaired listeners (Füllgrabe and Rosen, 2016). The Raven’s progressive matrices test is a nonverbal test of fluid intelligence (Nisbett et al., 2012), the ability to solve novel reasoning problems; this ability is associated with other constructs, including working memory (Kyllonen, Christal, 1990, Martínez, Burgaleta, Román, Escorial, Shih, Ángeles Quiroga, Colom, 2011), and processing speed (Kievit et al., 2016), although the relations between these constructs remain a matter of debate (Duncan et al., 2017).

The digit span tests were administered through a custom computerized interface that presented the sequences of digits to the participant through loudspeakers at a rate of one digit per second using the recordings of McShefferty et al. (2013). The sequences started at a length of two digits, and contained digits in the 1–9 range. They were the same for each participant and did not contain repeated digits up to a length of nine digits; if a participant was able to recall a sequence of nine digits, sequences with more than 10 digits were chosen by random sampling with replacement from the 1–9 range. The interface was controlled by the experimenter, who also input the participant’s responses on the computer.

The reading span task followed the format of Rönnberg et al. (1989). Participants were presented with sets of sentences of increasing length (3–6 sentences). The words composing each sentence were presented one at a time at a rate of 0.8 words per second. Sentences were separated by blank intervals of 1.75 seconds, during which participants had to say “yes” if the sentence made sense (e.g. “The captain saw his boat”), or “no”, if it did not make sense (e.g. “The train sang a song”). At the end of a set of sentences participants were prompted to recall either the first, or the last words of each sentence in the set (in any order). The test was scored on the total number of correctly recalled words (the task of judging whether each sentence is meaningful is a secondary task that is not typically scored).

The Raven’s progressive matrices test is a multiple-choice test in which participants have to select the missing piece in a geometric pattern. The full 60-item test was administered in accordance with the guidelines set out in the manual (Raven and Raven, 2003). The test was scored by counting the total number of correct responses.

### 2.8 Equipment

All auditory tests took place in double-walled soundproof booths (IAC Acoustics, Winchester, UK). Cognitive tests and questionnaires were completed in a quiet room. The stimuli for the psychophysical tests and audiometry were generated in Python (Python Software Foundation, Delaware, United States) with 32-bit resolution. The stimuli for the speech tests consisted of 16-bit WAV recordings. Stimuli were played through either an EMU 0204 USB sound card (E-MU Systems, Scotts Valley, U.S.A.), or a 24-bit RME Hammerfall DSP Multiface DAC (RME Intelligent Audio Solutions, Germany). Sennheiser HDA300 headphones (Sennheiser electronic GmbH & Co. KG, Hanover, Germany) were used for the measurement of audiometric thresholds. Sennheiser HDA650 headphones were used for all the other auditory tests.

### 2.9 Psychometric functions fitting

Thresholds for the psychophysical and speech-reception tasks were obtained by fitting psychometric functions on the data acquired with the UML procedure. Psychometric functions were fit via MCMC methods (Kuss et al., 2005), using a Logistic function. The midpoint, slope, and lapse rate were free parameters, while the guess rate was fixed at the reciprocal of the number of response alternatives. Normal (on a linear or log scale depending on the task) priors were used for the midpoint, while gamma priors were used for the parameter controlling the slope, and for the lapse rate. Priors for the midpoint and the slope were centered on average values of these parameters obtained via preliminary maximum likelihood fits (Wichmann and Hill, 2001). Further details of the fitting procedures are given in the supplementary materials.

### 2.10 Principal component analysis of cognitive tests scores

In order to reduce the number of predictor variables, the scores of the four cognitive tests were subject to principal component analysis (PCA; Sharma, 1996). The scores of each test were standardized by subtracting the sample mean and dividing by the sample standard deviation before being entered into the PCA. The eigenvalues and percentage of variance explained by the resulting principal components (PCs) are shown in Table S1, while the loadings of the components on each test are shown in Table S2. The first two components accounted for 74% of the variance (PC1: 50%, PC2: 24%), and were retained as predictor variables for further analyses. PC1 had correlations between 0.6 to 0.8 with all tests. The main loadings of PC2 were a positive correlation with the reading span task (0.6), and a negative correlation (-0.6) with the forward digit span task.

### 2.11 Statistical analyses

All analyses were performed using Bayesian models (Kruschke, 2014, McElreath, 2016, Ntzoufras, 2008) implemented by MCMC simulations using JAGS (Plummer, 2003) and R (R Core Team, 2020). For all MCMC simulations the chains for the main parameters of interest were monitored for convergence using trace plots, and where available the Gelman-Rubin statistic. The chains were also monitored for autocorrelation to ensure an effective sample size of at least ≃10,000 samples for the main parameters of interest (Kruschke, 2014).

The data were analyzed using robust mixed-effect multiple regression models (Gelman, Hill, 2007, Singmann and Kellen, 2019) which included both categorical and continuous predictors, as well as random effects of subjects. Robust regression uses a Student’s *t* distribution instead of a Normal distribution for describing residuals, minimizing the potential influence of outliers on the estimated regression coefficients (Kruschke, 2014). For categorical predictors an unweighted effect coding scheme was used (Aiken et al., 1991). Continuous variables were standardized using the Friedrich method (Aiken, West, Reno, 1991, Friedrich, 1982) before being entered into the analyses. Unstandardized coefficients corresponding to those resulting from an analysis of the mean-centered variables can be obtained by scaling using the appropriate standard deviation terms (Aiken et al., 1991). The priors for the slope coefficients in the models were set differently for coefficients that were of main interest in the analysis, and coefficients that were expected to affect the dependent variable, but were not of great analytical interest, such as the effect of masker location on CRM speech-reception threshold. For the latter effects, the priors were very broad on the scale of the data. Shrinkage priors were used for the former: the standardized coefficients were described by a *t* distribution centered at zero, with 1 degree of freedom, and scale parameter set to 0.1. This prior assumes that the standardized slope coefficients should be generally close to zero, where the narrow peak of the *t* distribution is located, reflecting a belief that effect sizes will be generally small. However, owing to its heavy tails the *t* prior can accommodate coefficients much larger than zero if the likelihood provides clear evidence for this (Kruschke, 2014). The interpretation of the standardized slope coefficients, and hence of the priors set on them, differs for continuous and categorical variables. For continuous variables the standardized slope coefficient is the change of the dependent variable in standard deviation (sd) units, for a 1-sd change of the dependent variable. Categorical variables were not standardized, and the coefficients represent the shift in the value of the dependent variable (which was still set in sd units in our models) for the categorical level coded as 1, from the unweighted grand mean of the dependent variable over all the levels. Further details of the models are given in the supplementary materials, and the model code is available at https://doi.org/10.17605/OSF.IO/B69DS.

Effects were summarized by 99% credibility intervals (CIs) of the posterior distribution of the parameter of interest. These indicate that, according to the model, the parameter has a 99% probability of being enclosed within the bounds of the interval. The use of CIs to summarize the results of the study is in line with calls from different schools of statistical thought for a shift from crude null hypothesis testing to explicit estimation of the size of parameters of interest, and the uncertainty of these estimates (Gardner and Altman, 1986, Kruschke and Liddell, 2018, McShane, Gal, Gelman, Robert, Tackett, 2019, Schmidt, 1996). This approach emphasizes the idea that statistical results provide graded evidence, or different degrees of (un)certainty regarding a hypothesis, avoids conflating statistical significance with practical and/or theoretical significance, and acknowledges that single studies can rarely provide on their own conclusive evidence for or against an effect. Nonetheless, it is difficult to summarize succinctly the results of a large-scale study without making some categorical statements. For this reason we will refer to parameters whose 99% CIs excludes zero as being credibly different from zero^3^ to highlight the most salient findings, but we will also emphasize the size and uncertainty of effect estimates.

#### 2.11.1 Amplitude modulation detection model

AM detection thresholds, measured in dB, were modeled jointly across stimulus levels and modulation frequencies. Predictor variables included age, pure tone audiometric thresholds at 2 kHz (PT_2_), log_10_TCNE, the first (Cog1) and second (Cog2) principal components extracted from the PCA on cognitive test scores, and the number of years of musical practice. Because this latter variable was right skewed, a cube root transformation was applied to it before statistical analyses. For brevity the cube-root-transformed number of years of musical practice will be referred to as MUS. Stimulus level (40 or 80 dB SPL), modulation frequency (25, 50, or 100 Hz), and the interactions of these two variables with age, PT_2_, and log_10_TCNE were also included as predictor variables. All terms for the AM detection model are listed in Table S7.

#### 2.11.2 Frequency discrimination model

Log-transformed frequency-discrimination thresholds (measured in % frequency difference) were modeled jointly across pure tone levels and frequencies. Predictor variables included age, pure tone audiometric thresholds at 0.5 kHz (PT_0.5_) for the 0.6-kHz frequency-discrimination data, and PT_2_ for the 2-kHz frequency-discrimination data, log_10_TCNE, Cog1, Cog2, and MUS. Stimulus level (40 or 80 dB SPL), frequency (0.6 or 2 kHz), and the interactions of these two variables with age, pure tone audiometric thresholds, and log_10_TCNE were also included as predictor variables. All terms for the frequency-discrimination model are listed in Table S8.

#### 2.11.3 F0 discrimination model

Log-transformed F0-discrimination thresholds (measured in % F0 difference) were modeled jointly across complex tone levels. Predictor variables included age, PT_2_, log_10_TCNE, Cog1, Cog2, and MUS. Stimulus level (40 or 80 dB SPL), and the interactions of this variable with age, pure tone audiometric thresholds, and log_10_TCNE were also included as predictor variables. All terms for the F0-discrimination model are listed in Table S9.

#### 2.11.4 Interaural phase difference detection with AM tones model

Log-transformed IPD detection thresholds (measured in degrees) were modeled jointly across amplitude-modulated tones carrier frequencies and levels. Predictor variables included age, PT_0.5_ for the amplitude-modulated tone with a 0.6-kHz carrier, and PT_2_ for the amplitude-modulated tone with a 2-kHz carrier, log_10_TCNE, Cog1, Cog2, and MUS. Stimulus level (40 or 80 dB SPL), carrier frequency (0.6 or 2 kHz), and the interactions of these two variables with age, pure tone audiometric thresholds, and log_10_TCNE were also included as predictor variables. All terms for the MOD-IPD detection model are listed in Table S10.

#### 2.11.5 Interaural phase difference detection model with pure tones model

Log-transformed IPD detection thresholds (measured in degrees) were modeled jointly across tone levels. Predictor variables included age, PT_0.5_, log_10_TCNE, Cog1, Cog2, and MUS. Stimulus level (40 or 80 dB SPL), and the interactions of this variable with age, PT_0.5_, and log_10_TCNE were also included as predictor variables. All terms for the PT-IPD detection model are listed in Table S11.

#### 2.11.6 Coordinate response measure model

Speech-reception thresholds, measured in dB, were modeled jointly across maskers levels and offsets relative to the target speech. Predictor variables included age, average pure tone audiometric thresholds from 0.125 to 2 kHz (PTA_0.125–2_), log_10_TCNE, Cog1, Cog2, and MUS. Maskers level (42 or 77 dB SPL), maskers offset (colocated or offset), and the interactions of these two variables with age, PTA_0.125–2_, and log_10_TCNE were also included as predictor variables. Additionally the model included interaction terms between maskers offset and Cog1, Cog2, and MUS. All terms for the CRM model are listed in Table S12.

#### 2.11.7 Digit triplets test model

Speech-reception thresholds, measured in dB, were modeled jointly across masker levels. Predictor variables included age, PTA_0.125–2_, log_10_TCNE, Cog1, Cog2, and MUS. Masker level (45 or 80 dB SPL) and the interactions of this variable with age, PTA_0.125–2_, and log_10_TCNE were also included as predictor variables. All terms for the DTT model are listed in Table S13.

#### 2.11.8 Musical intervals ratings model (consonance preference)

The standardized ratings (averaged across root notes) given to the dyads were modeled jointly across musical intervals and dyad levels. Predictor variables included age, PTA_0.125–2_, log_10_TCNE, Cog1, Cog2, and MUS. Dyad level (40 or 80 dB SPL), interval (TT or P5), and the interactions of these two variables with age, PTA_0.125–2_, and log_10_TCNE were also included as predictor variables. Additionally, the model included interaction terms between interval and Cog1, Cog2, and MUS. All terms for the musical interval ratings model are listed in Table S14.

#### 2.11.9 Speech, spatial, and qualities of hearing scale model

The dependent variable consisted of the SSQ12 scores, averaged across questions. Predictor variables included age, PTA_0.125–2_, average pure tone thresholds between 4 and 12 kHz (PTA_4–12_), log_10_TCNE, Cog1, Cog2, and MUS. All terms for the SSQ12 model are listed in Table S15.

## 3 Results

### 3.1 Predictor variables

**Fig. 1:**
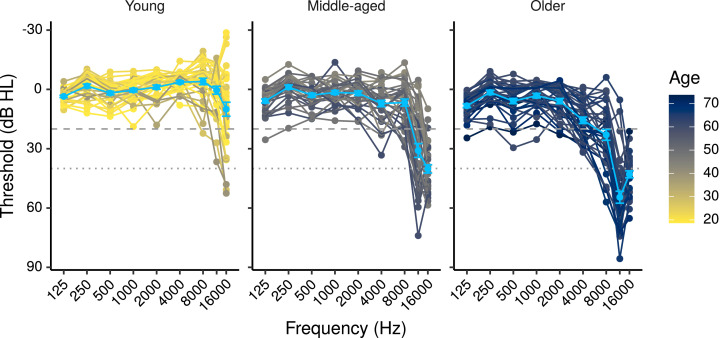
Audiometric thresholds for the study participants. The light blue points plot mean thresholds ± 1 standard error of the mean (s.e.m.) for each age group. The dashed and dotted lines mark respectively 20, and 40 dB HL.

Fig. 1 shows the audiometric thresholds for the participants included in the study, while Fig. S2 shows the other predictors (log_10_TCNE, MUS, Cog1, and Cog2) as a function of age. Tables 1 and 2 show correlations among predictor variables used in this study, along with 99% CIs computed with a Bayesian model based on that of Lee and Wagenmakers (2014, chap. 5, see supplementary materials for details). Not all of the predictors shown in the tables were used in all models, for details of the predictors used in each model see Section 2.11.

**Table 1:** Matrix of correlations (r) among the predictor variables. 99% CIs are shown in brackets. Only the correlations between some of the predictor variables are shown in this table. The remaining correlations are shown in Table 2. Some entries are repeated across the two tables.

	PT_0.5_	PT_2_	Log_10_TCNE	Music $\sqrt[{3}]{\text{Y}}$	Cog1	Cog2
Age	0.26 (0.01 – 0.48)	0.44 (0.21 – 0.62)	0.02 (–0.23 – 0.28)	0.10 (–0.16 – 0.34)	–0.06 (–0.30 – 0.20)	–0.28 (–0.50 – -0.04)
PT_0.5_		0.41 (0.18 – 0.60)	–0.22 (–0.44 – 0.03)	–0.01 (–0.26 – 0.24)	–0.13 (–0.37 – 0.13)	–0.12 (–0.36 – 0.13)
PT_2_			–0.02 (–0.27 – 0.23)	–0.03 (–0.29 – 0.21)	–0.16 (–0.41 – 0.09)	–0.14 (–0.38 – 0.12)
Log_10_TCNE				–0.01 (–0.26 – 0.25)	0.05 (–0.20 – 0.30)	0.07 (–0.19 – 0.31)
Music $\sqrt[{3}]{\text{Y}}$					0.07 (–0.20 – 0.31)	0.20 (–0.05 – 0.43)

**Table 2:** Matrix of correlations (r) among the predictor variables. 99% CIs are shown in brackets. Only the correlations between some of the predictor variables are shown in this table. The remaining correlations are shown in Table 1. Some entries are repeated across the two tables.

	PTA_0.125-2_	PTA_4__–__12_	Log_10_TCNE	Music $\sqrt[{3}]{\text{Y}}$	Cog1	Cog2
Age	0.39 (0.15 – 0.58)	0.84 (0.74 – 0.90)	0.02 (–0.23 – 0.28)	0.10 (–0.16 – 0.34)	–0.06 (-0.30 – 0.20)	–0.28 (–0.50 – –0.04)
PTA_0.125-2_		0.50 (0.30 – 0.68)	–0.14 (–0.39 – 0.12)	–0.06 (–0.30 – 0.19)	–0.19 (-0.43 – 0.06)	–0.16 (–0.40 – 0.08)
PTA_4__–__12_			0.06 (-0.21 – 0.29)	–0.06 (–0.31 – 0.19)	–0.01 (-0.25 – 0.24)	–0.30 (–0.51 – –0.06)

Correlations between the predictor variables were all low or moderate, with the exception of the correlation between age and PTA_4–12_, two variables that were used together only in the SSQ12 model. As expected there were also credible low/moderate correlations between age and audiometric thresholds at other frequencies or frequency averages (and between audiometric thresholds at different audiometric frequencies). There were credible negative correlations between age and Cog2, and between PTA_4–12_ and Cog2.

It is notable that, in this sample, age was not associated with increased log_10_TCNE. This may reflect geographical or historical peculiarities of the participants sample, as well as the fact that they were a self-selected sample of volunteers from the general population. For most listeners the major contributor to log_10_TCNE was recreational noise exposure, and this tended to be concentrated in their youth years. Nonetheless TCNE had a large spread across the sample, varying over more than three orders of magnitude.

### 3.2 Amplitude modulation detection

**Fig. 2:**
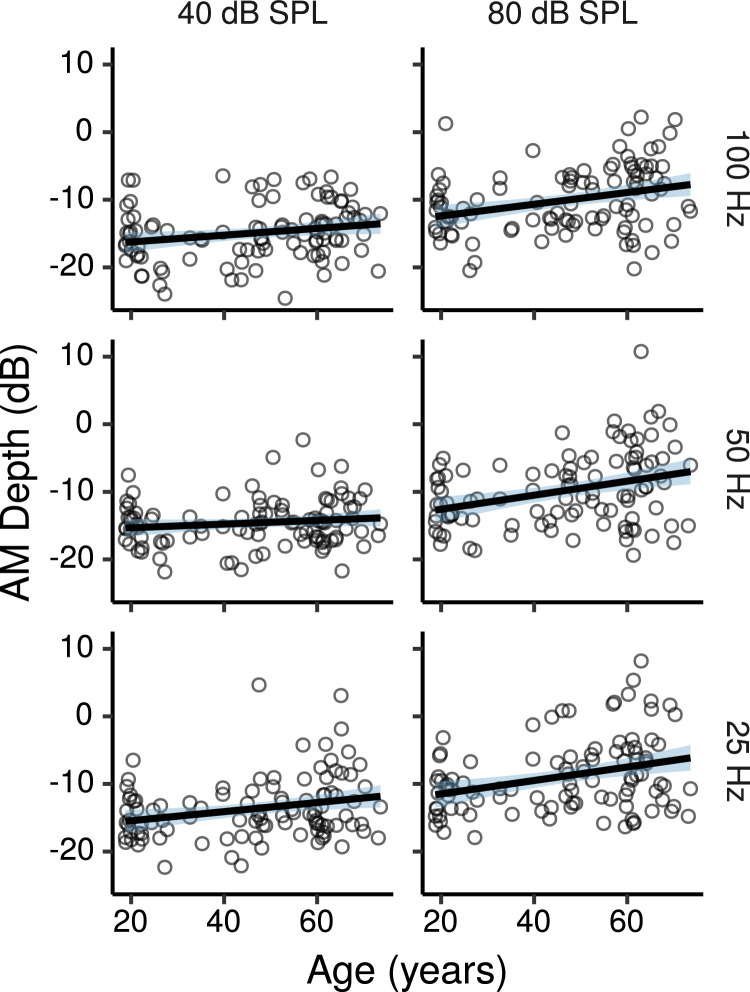
AM detection thresholds [20log(m)] by age. Each panel shows a least squares line fit of threshold by age with 95% confidence intervals as a visual aid. The slope for the effect of age estimated by the Bayesian multiple regression model is not the same as that shown in the figure.

Fig. 2 shows the AM detection thresholds as a function of age for each modulation frequency and stimulus level. Thresholds were generally within the range of possible AM depths, but a few participants (mostly older) had great difficulty in some conditions of this task, so that their thresholds estimated by fitting psychometric functions were above the range of possible AM depths (m>1, or equivalently AM depth in dB >0; see supplementary materials for details of the psychometric function fitting procedures).

**Fig. 3:**
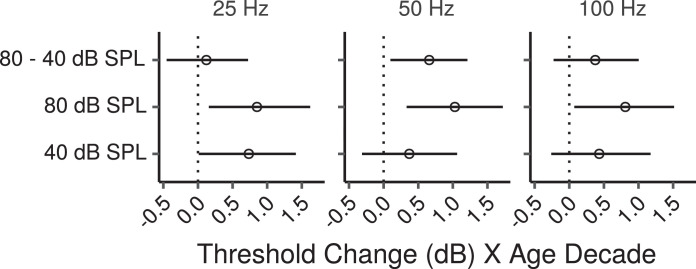
Posterior medians (circles) and 99% CIs for the effects of age on AM detection thresholds estimated by the Bayesian multiple regression model. Effects are plotted as threshold change for an age increase of 10 years.

Fig. 3 shows the effects of age on AM detection thresholds estimated by the multiple regression model at each modulation frequency, and stimulus level, as well as the differential effect of age between the high and low stimulus levels. There were trends for thresholds to increase with age in all conditions, with posterior medians of ∼0.5 – 1 dB per age decade, although these increases were not credibly different from zero for the 50 and 100 Hz modulation frequencies at the low stimulus level. For the other conditions the CIs, which provide a measure of the uncertainty of effect estimates, were compatible with effects ranging in size from ∼ 0.1 to 1.5 dB per age decade.

The differential effect of age between the high and low stimulus level was credibly different from zero only for the 50 Hz modulation frequency, with thresholds increasing ∼ 0.7 dB (CI: 0.1 – 1.2) more per age decade for the high compared to the low stimulus level. A trend in the same direction was present for the stimulus with a 100 Hz modulation frequency, while for the 25-Hz stimulus there was no evidence of larger age-related effects for the high compared to the low stimulus level.

Interestingly, AM detection thresholds appeared to be lower overall at 40 than at 80 dB SPL. This was the case also for young participants, with the model estimating thresholds at 80 dB SPL 3.7 dB (CI: 2.6 – 4.7) higher than at 40 dB SPL at age 20 (with predictors other than age set at their mean). Previous studies of AM detection have generally found that thresholds decrease as the stimulus level increases (Kohlrausch, 1993, Moore, Glasberg, 2001), an effect attributed to spread of excitation that can improve detection by allowing listeners to combine information across multiple auditory filters, and/or by allowing listeners to detect the modulation at off-frequency places that are less subject to basilar-membrane compression (Kohlrausch et al., 2000). Both these effects favoring AM detection at high stimulus levels would have been reduced or eliminated in the current study due to the presence of the highpass noise masker. Additionally, the small decrement in performance at the high compared to the low stimulus level found in the current study may be due to masking of the AM tone by the lower noise band, which would have had a larger upward spread at 80 than at 40 dB SPL.

The effects of PT_2_ and log_10_TCNE are shown in Figures S3 and S4, respectively. None of these effects was credibly different from zero. The effects of Cog1, Cog2, and MUS are listed in Table S3. The effect of Cog1 was credibly different from zero, with greater Cog1 scores associated with smaller thresholds (CI: -2.1 – -0.2). The effects of Cog2 and MUS were not credibly different from zero.

### 3.3 Frequency and F0 discrimination

**Fig. 4:**
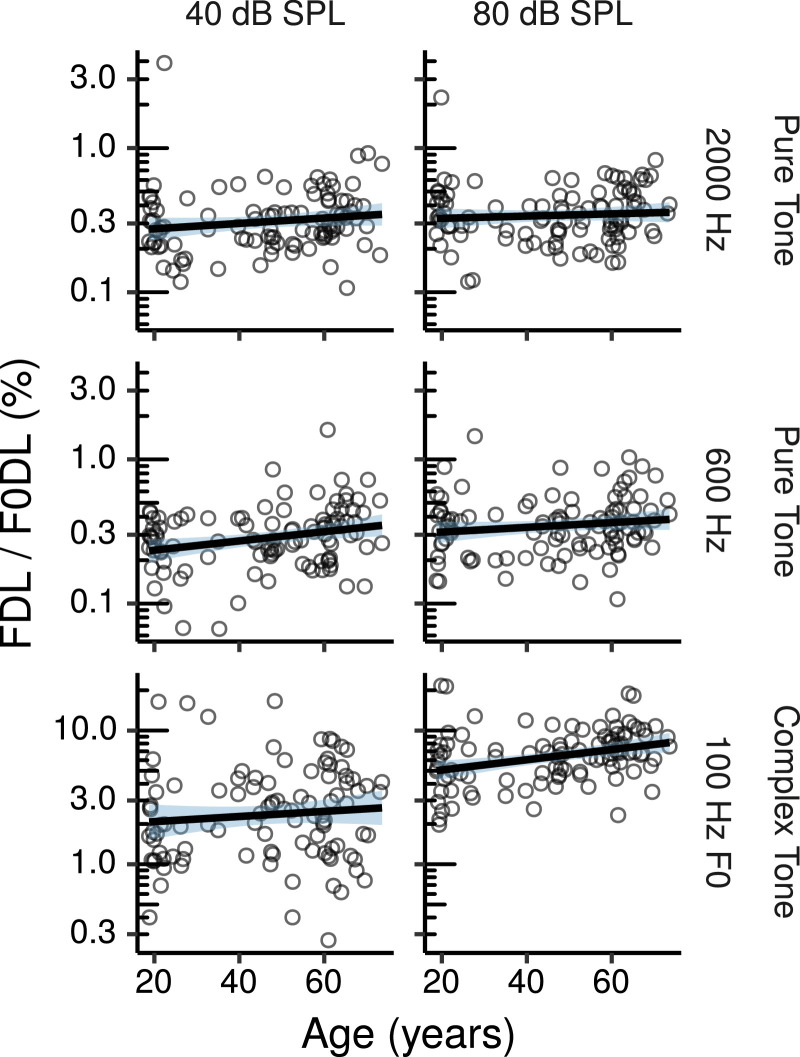
Frequency and F0 discrimination thresholds by age. Each panel shows a least squares line fit of threshold by age with 95% confidence intervals as a visual aid. The slope for the effect of age estimated by the Bayesian multiple regression model is not the same as that shown in the figure.

**Fig. 5:**
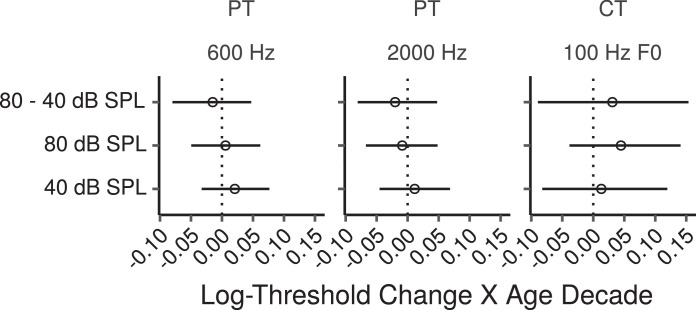
Posterior medians (circles) and 99% CIs for the effects of age on pure tone (PT) frequency discrimination thresholds, and complex tone (CT) F0 discrimination thresholds estimated by the Bayesian multiple regression models. Effects are plotted as threshold change for an age increase of 10 years.

Fig. 4 shows the frequency and F0 discrimination thresholds as a function of age. The effects of age estimated by the multiple regression models are shown in Fig. 5. None of these effects was credibly different from zero. The log-threshold changes shown in Fig. 5 can be converted to proportional changes by exponentiation. When expressed in this way the CIs for the effects of age on pure tone frequency discrimination were relatively narrow, and compatible with increases of at most a factor of ∼ 1.1 per age decade. The CIs for the differential effects at the high compared to the low stimulus level were also relatively narrow, and compatible with a greater age effect for the 80 dB SPL stimulus of at most a factor of 1.05 per age decade.

The CIs for the effects of age on F0 discrimination for the unresolved complex tone were somewhat larger, and compatible with an age-related increase of a factor of ∼ 1.15 per age decade, and a greater age effect for the 80 dB SPL stimulus of at most a factor of 1.16 per age decade.

The effects of audiometric thresholds and log_10_TCNE are shown in Figures S5 and S6, respectively. The effects of audiometric thresholds on pure tone frequency discrimination were all credibly greater than zero, except for the 80-dB SPL 0.6 kHz pure tone, that nonetheless showed a similar trend of worse discrimination thresholds with larger audiometric losses. The CIs for the credible effects ranged from a threshold increase of a factor of ∼ 1.01 to 1.4 per 10 dB of audiometric threshold increase. The effects of audiometric thresholds on complex tone F0 discrimination were not credibly different from zero. There were no credible effects of log_10_TCNE on frequency or F0 discrimination. The CIs for the effects of log_10_TCNE were relatively narrow for pure tone frequency discrimination (compatible with threshold increases of at most a factor of 1.17 per tenfold increase in noise exposure in one condition), while they were somewhat larger for complex tone F0 discrimination (compatible with threshold increases of at most a factor of 1.27 per tenfold increase in noise exposure).

The effects of Cog1, Cog2, and MUS are listed in Table S3. None of these effects were credibly different from zero, although there were trends for better pure tone frequency discrimination thresholds for individuals with higher cognitive test scores and greater musical experience, and for better F0 discrimination thresholds with increases in Cog2.

### 3.4 Interaural phase difference detection

**Fig. 6:**
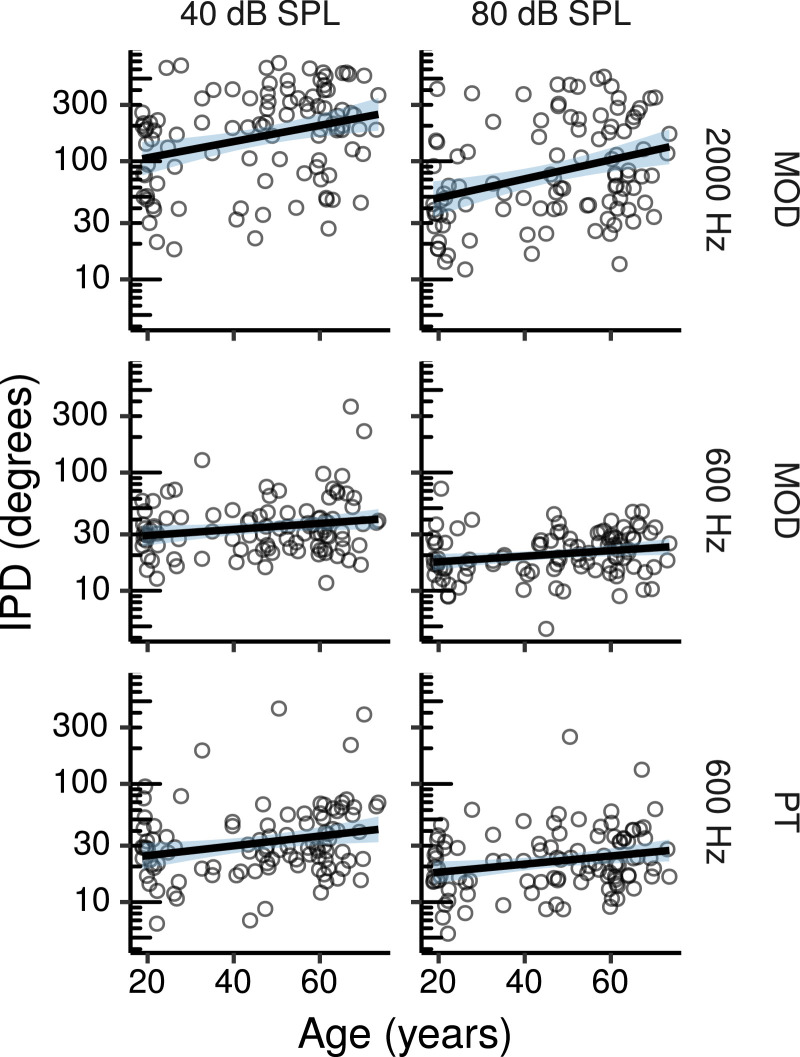
IPD detection thresholds by age. MOD refers to conditions in which the IPD was applied to the modulator of an AM tone, and PT to conditions in which the IPD was applied to a pure tone. Each panel shows a least squares line fit of threshold by age with 95% confidence intervals as a visual aid. The slope for the effect of age estimated by the Bayesian multiple regression model is not the same as that shown in the figure.

**Fig. 7:**
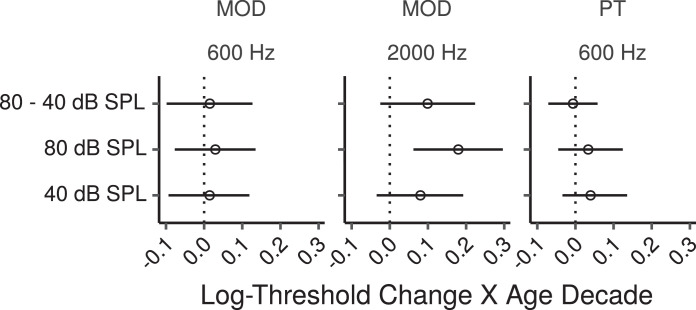
Posterior medians (circles) and 99% CIs for the effects of age on MOD and PT IPD detection thresholds estimated by the Bayesian multiple regression models. MOD refers to conditions in which the IPD was applied to the modulator of an AM tone, and PT to conditions in which the IPD was applied to a pure tone. Effects are plotted as threshold change for an age increase of 10 years.

Fig. 6 shows thresholds for MOD and PT IPD detection as a function of age. The CIs for age effects estimated by the multiple regression model are shown in Fig. 7.

Age effects for MOD IPD detection at 600 Hz were not credibly different from zero, with posterior medians close to zero, and CIs compatible with either increases or decreases of a factor of ∼ 1.1 per age decade. For MOD IPD detection at 2000 Hz there was a credible age-related increase in thresholds at 80 dB SPL, with the CI compatible with threshold increases ranging from a factor of ∼ 1.1 to ∼ 1.3 per age decade. A trend in the same direction was also present at 40 dB SPL (CI: 0.97 – 1.21 factor change per age decade). The effect tended to be larger at 80 compared to 40 dB SPL, but the effect difference was not credibly larger than zero (CI: 0.98 – 1.25 factor change per age decade). No credible effects of age were present for PT IPD detection with CIs compatible with age-related changes of a factor of ∼ 0.96 to a factor of ∼ 1.15 per age decade.

The effects of audiometric thresholds are shown in Figure S7. There were credible effects of audiometric thresholds on MOD IPD detection at 40 dB SPL, with CIs compatible with threshold increases of a factor of just above 1 to a factor of 1.7 per 10-dB increase in audiometric thresholds. A trend in the same direction and of similar magnitude was present for PT IPD detection (CI: 0.99 – 1.6 factor change per 10-dB increase in audiometric thresholds). Effects at 80 dB SPL were not credibly different from zero for either MOD or PT thresholds (CIs ranging from ∼ 0.7 to ∼ 1.4 factor change per 10-dB audiometric threshold increase).

Figure S8 shows the effects of log_10_TCNE. None of these effects were credibly different from zero, with posterior median effects close to zero (factor change close to 1), and CIs ranging from a factor change of ∼ 0.8 to ∼ 1.2 per tenfold increase in noise exposure.

The effects of Cog1, Cog2, and MUS are listed in Table S3. None of these effects were credibly different from zero, although there were trends for better MOD and PT IPD detection thresholds for individuals with higher cognitive test scores.

### 3.5 Coordinate response measure

**Fig. 8:**
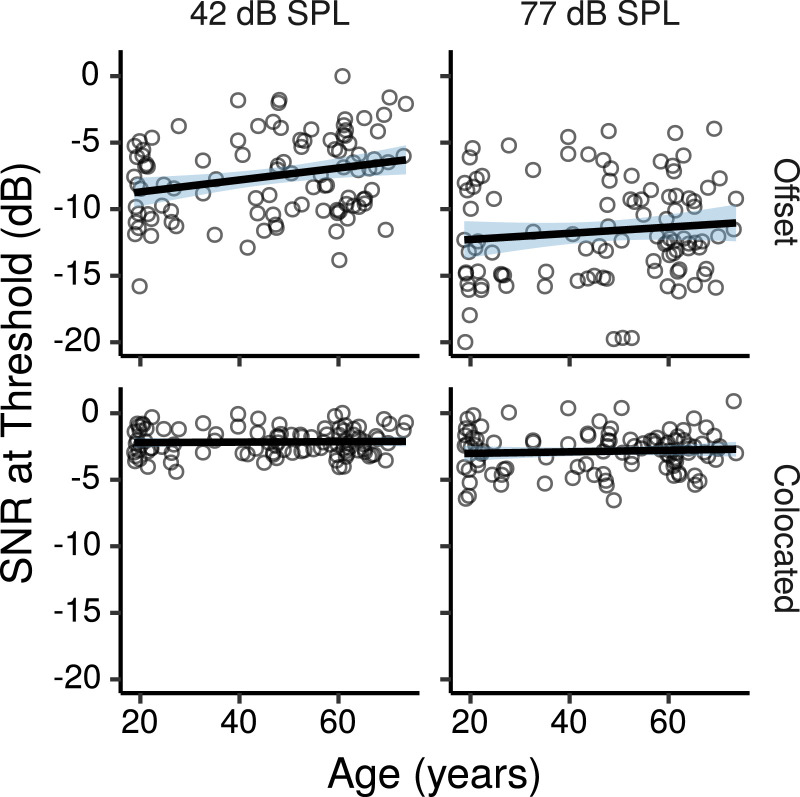
CRM thresholds by age. Each panel shows a least squares line fit of threshold by age with 95% confidence intervals as a visual aid. The slope for the effect of age estimated by the Bayesian multiple regression model is not the same as that shown in the figure.

**Fig. 9:**
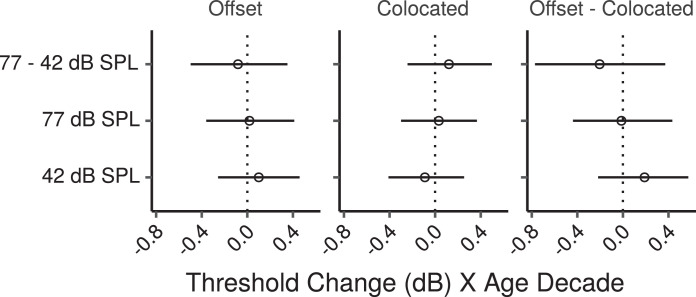
Posterior medians (circles) and 99% CIs for the effects of age on CRM speech reception thresholds estimated by the Bayesian multiple regression model. Effects are plotted as threshold change for an age increase of 10 years. The leftmost, and the central panel show effects for the conditions with maskers that were either offset, or colocated with the target, respectively. The rightmost panel shows the effect difference between these two conditions. Values above zero in this panel would indicate that spatial release from masking decreases with increasing age.

Fig. 8 shows the speech-reception thresholds in the CRM task as a function of age. The effects of age estimated by the multiple regression model for the offset and colocated conditions are shown in Fig. 9. None of these effects were credibly different from zero, with posterior medians close to zero, and CIs consistent with age-related changes in either direction of less than 0.5 dBs per age decade. Fig. 9 also shows the effect differences between the colocated and offset conditions, which provides a measure of age-related changes in spatial release from masking. There were no credible effects of age on spatial release from masking.

The effects of PTA_0.125–2_ on CRM thresholds are shown in Figure S9. In the offset conditions there were credible CRM threshold increases with increasing PTA_0.125–2_ both at 42 dB SPL (CIs: 1.5 – 4.1 dBs per 10-dB PTA_0.125–2_ increase) and at 77 dB SPL (CI: 0.3 – 3.1 dBs per 10-dB PTA_0.125–2_ increase). Although the effect tended to be larger at 42 than at 77 dB SPL, this difference was not credibly different from zero (CI: -2.5 – 0.3 dBs per 10-dB PTA_0.125–2_ increase). There were no credible effects of PTA_0.125–2_ in the colocated condition. Spatial release from masking decreased with increasing PTA_0.125–2_ both at 42 dB SPL (CI: 0.7 – 3.5 dBs per 10-dB PTA_0.125–2_ increase), and at 77 dB SPL (CI: 0.4 – 3.4 dBs per 10-dB PTA_0.125–2_ increase).

The effects of log_10_TCNE are shown in Figure S10. None of these effects was credibly different from zero. The CIs were consistent with effects in either direction of at most ∼ 1 dB threshold change per tenfold increase in noise exposure. There were no credible effects of log_10_TCNE on spatial release from masking.

The effects of Cog1, Cog2, and MUS are listed in Table S3. None of these effects were credibly different from zero, although there were trends for better thresholds for individuals with higher cognitive test scores both in the offset and colocated conditions. In the offset conditions there was also a trend for better thresholds for individuals with greater musical experience.

### 3.6 Digit triplets test

**Fig. 10:**
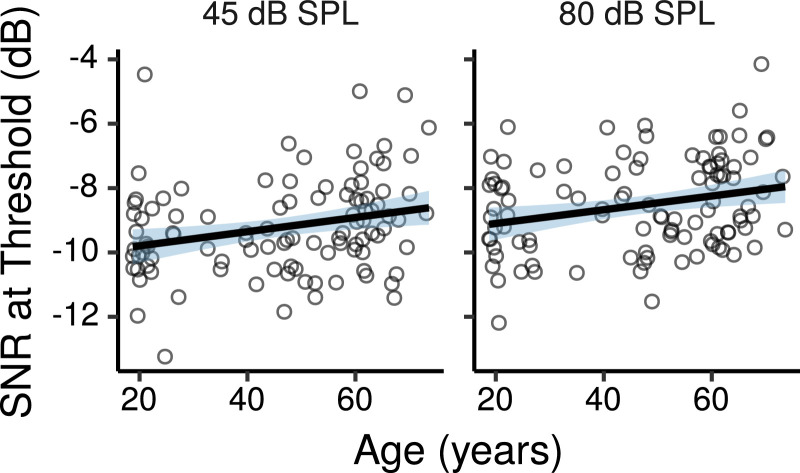
DTT thresholds by age. Each panel shows a least squares line fit of threshold by age with 95% confidence intervals as a visual aid. The slope for the effect of age estimated by the Bayesian multiple regression model is not the same as that shown in the figure.

**Fig. 11:**
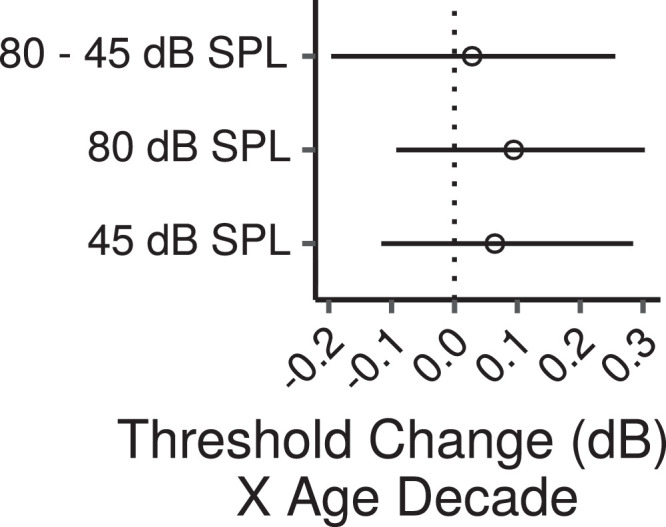
Posterior medians (circles) and 99% CIs for the effects of age on DTT speech reception thresholds estimated by the Bayesian multiple regression model. Effects are plotted as threshold change for an age increase of 10 years.

Fig. 10 shows the speech-reception thresholds in the DTT as a function of age. The effects of age estimated by the multiple regression model are shown in Fig. 11. These were not credibly different from zero, although there were trends for small threshold increases with increasing age at both levels, with CIs compatible with threshold changes of ∼ -0.1 to 0.3 dB per age decade.

The effects of PTA_0.125–2_ on DTT thresholds are shown in Figure S11. There was a credible increase in DTT thresholds with increasing PTA_0.125–2_ at 45 dB SPL (CI: 0.1 – 1.6 dB per 10-dB PTA_0.125–2_ increase), and a trend in the same direction at 80 dB SPL (CI: -0.3 – 1.1 dB per 10-dB PTA_0.125–2_ increase).

Figure S12 shows the effects of log_10_TCNE on DTT thresholds. These effects were not credibly different from zero at either stimulus level, with CIs ranging from ∼ -0.3 to ∼ 0.4 dB per tenfold increase in noise exposure.

The effects of Cog1, Cog2, and MUS are listed in Table S3. None of these effects were credibly different from zero, although there were trends for better DTT thresholds for individuals with higher cognitive test scores, and for worse DTT thresholds for individuals with greater musical experience.

### 3.7 Consonance preference

**Fig. 12:**
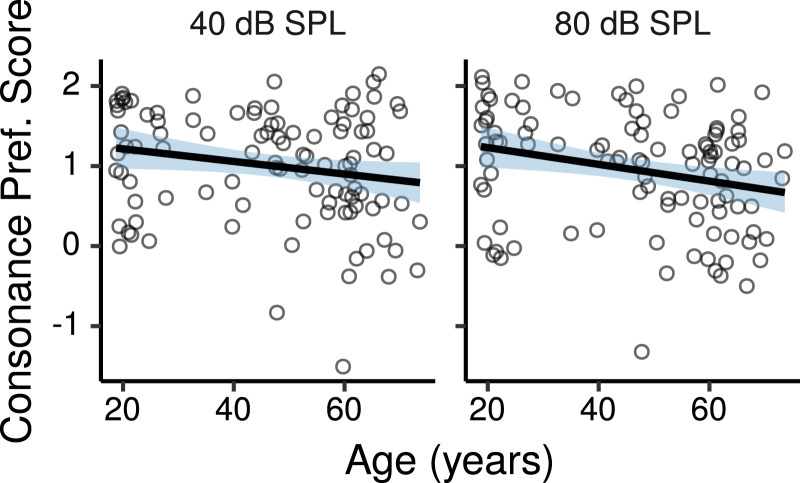
Consonance preference scores by age. Each panel shows a least squares line fit of consonance preference scores by age with 95% confidence intervals as a visual aid. The slope for the effect of age estimated by the Bayesian multiple regression model is not the same as that shown in the figure.

Fig. 12 shows the consonance preference scores (ratings for the consonant perfect fifth interval minus ratings for the dissonant tritone interval) as a function of age (ratings for each interval are shown in Figure S13).

**Fig. 13:**
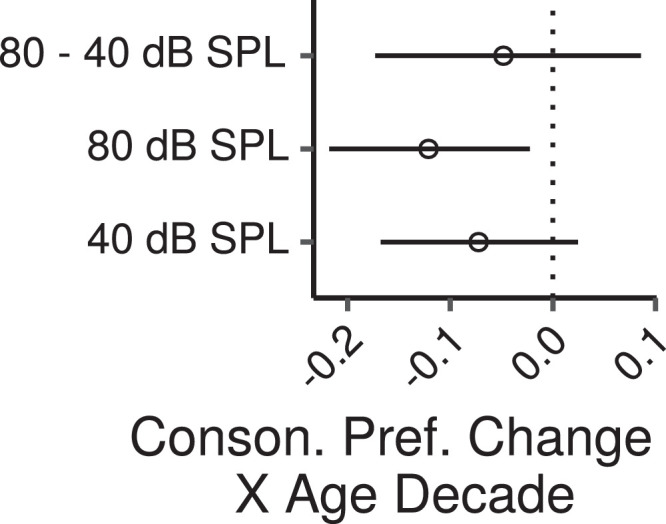
Posterior medians (circles) and 99% CIs for the effects of age on consonance preference scores estimated by the Bayesian multiple regression model. Effects are plotted as score change for an age increase of 10 years.

Fig. 13 shows the effects of age on consonance preference scores estimated by the multiple regression model. There was a credible decrease in consonance preference scores with increasing age at 80 dB SPL (CI: -0.21 – -0.02 per age decade) and a trend in the same direction at 40 dB SPL (CI: -0.17 – 0.02 per age decade). The effect of age was not credibly different between the two levels (CI: -0.17 – 0.09).

There were no credible effects of PTA_0.125–2_ on consonance preference scores (Figure S14), with CIs compatible with maximal score changes of ∼±0.3 per 10-dB PTA_0.125–2_ increase.

The effects of log_10_TCNE were also not credibly different from zero (Figure S15) at either level (CI 40 dB SPL: -0.14 – 0.23; CI 80 dB SPL: -0.10 – 0.31 per tenfold increase in noise exposure).

CIs for the effects of Cog1, Cog2, and MUS are listed in Table S3. There was a credible increase in consonance preference scores with increasing Cog1 scores (CI: 0.03 – 0.27), and a trend in the same direction for Cog2 (CI: -0.06 – 0.18). There was also a credible increase in consonance preference scores with MUS (CI: 0.05 – 0.26).

### 3.8 Speech, spatial, and qualities of hearing scale

**Fig. 14:**
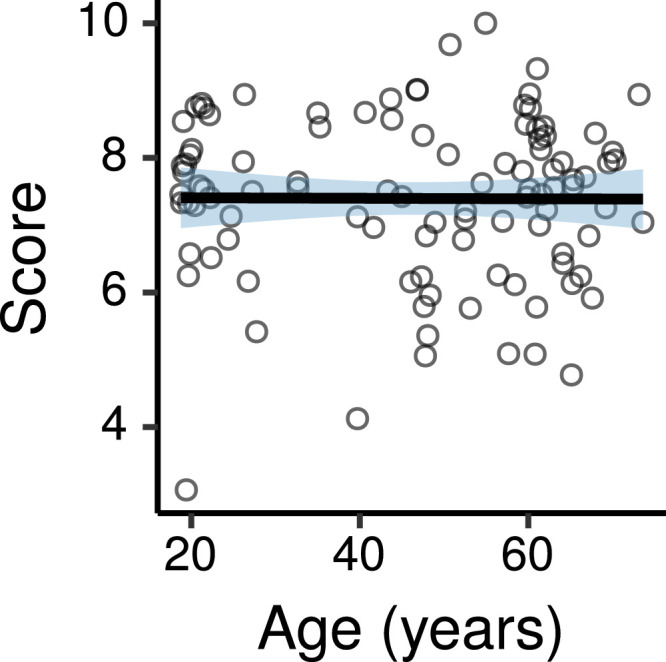
SSQ12 scores by age. The panel shows a least squares line fit of SSQ12 score by age with 95% confidence intervals as a visual aid. The slope for the effect of age estimated by the Bayesian multiple regression model is not the same as that shown in the figure.

Fig. 14 shows the average across-questions SSQ12 scores by age (scores for each SSQ12 question are shown in Figure S16). There were no credible effects of age (CI: -0.15 – 0.22 per age decade), PTA_0.125–2_ (CI: -0.7 – 0.3 per 10-dB PTA_0.125–2_ increase), PTA_4–12_ (CI: -0.2 – 0.16 per 10-dB PTA_4–12_ increase), or log_10_TCNE (CI: -0.37 – 0.23 per tenfold increase in noise exposure). Posterior medians for these effects were in each case close to zero, and the CIs were relatively narrow. There were no credible effects of Cog1, Cog2, or MUS (see Table S3 for the CIs).

## 4 Discussion

In this study we assessed the performance of 102 listeners ranging in age from 19 to 74 years on a range of auditory tasks that included several psychophysical tests thought to be dependent on auditory temporal processing, and speech reception in noise tests. For each task, performance was assessed both at a low and at a high stimulus level to test for specific effects of age-related CS, which was hypothesized to affect mainly performance at high stimulus levels. Performance in several tasks was negatively affected by age even after partialing out the effects of audiometric thresholds, lifetime noise exposure, cognitive abilities, and musical experience. However, for only one out of 13 tests were age-related declines credibly larger at the high than at the low stimulus level. Overall these results do not provide much evidence that age-related CS has substantial effects on psychophysical measures of auditory temporal processing or on speech-reception thresholds.

### 4.1 Differential high-low level effects

Age-related declines in the current study were credibly larger for AM detection at a high than at a low stimulus level for a tone amplitude-modulated at a rate of 50 Hz. No credible effects of age on the differential high-low level measure of performance were observed for the same tone modulated at rates of 25 or 100 Hz, although a weak trend in that direction was present for the 100-Hz modulated tone. There are three possible scenarios regarding this effect: i) it may be a genuine effect indicative of age-related CS affecting mainly L/M-SR fibers; ii) it may be a genuine effect, but unrelated to CS; iii) it may be a fortuitous result due to sampling error. We will consider each possibility in light of other available evidence from the current study and from previous studies in the literature.

If the differential age effect for the 50-Hz amplitude-modulated tone is a genuine effect reflecting CS, there should be an explanation for why it was detected only for this particular condition of the AM detection task, and not for the other conditions or for the other tasks of the current study. Further analyses of the posterior estimates do not provide strong evidence of specificity of this effect with respect to modulation rate because the 50-Hz effect was not credibly larger than the 25-Hz effect (CI: -0.23 – 1.33) or the 100-Hz effect (CI: -0.53 – 1.1), although there is at least trend for larger differential effects at the two higher modulation rates. A previous study (Grose et al., 2019) failed to find evidence of differential age effects at low and high stimulus levels on an AM detection task using a modulation rate of 80 Hz, but the sample size was small, so the study could have easily missed differential age effects. Another study (Prendergast et al., 2019) that found a differential effect in the opposite direction of that found in the current study for the 50-Hz AM, used a modulation rate of 25 Hz. The evidence from the current and previous studies reviewed above is to a certain extent consistent with the possibility of greater CS effects at higher modulation rates (in the 50–100 Hz range). However, if CS effects were greater at these higher stimulus rates, it is not clear why they were not detected in other psychophysical tasks of the current study (frequency/F0 discrimination and IPD detection) that used envelope or fine-structure modulation rates of 100 Hz or higher. Overall, while it cannot be excluded that the differential effect at 50 Hz reflects CS, the evidence for this hypothesis in light of other findings from the current and previous studies appears weak.

A second possible scenario is that the differential effect of age at high and low stimulus levels for 50-Hz AM detection is genuine, but is due to factors other than CS. Although we do not have specific suggestions on other mechanisms that could explain this effect, this scenario would fit better with the rest of the available data that do not provide evidence of CS effects for other tasks. Finally, although we strove to use models that would minimize the likelihood of false detections, and although our sample size was rather large, it cannot be excluded that the differential age effect for 50-Hz AM detection is due to sampling error. This possibility is rarely discussed in scientific writings, but no statistical method, Bayesian or Frequentist, can guarantee that a particular effect is not due to sampling error.

Besides 50-Hz AM detection the only other task showing a consistent trend towards a differential effect of age at high and low stimulus levels was IPD MOD detection at 2 kHz. Similarities between these two tasks include the use of a 2-kHz carrier frequency, and the fact that both tasks involve encoding envelope periodicities. To a large extent they share these similarities with the F0 discrimination task, for which there was no obvious trend for a differential effect of age at high and low stimulus levels, although the CIs for that effect are relatively large and would be compatible with such an effect. Overall, while we do not think that the current data provide much evidence for differential age effects at high and low stimulus levels, tasks involving the detection of envelope periodicities may be good candidates for further investigations of such effects.

The lack of credible differential effects of age at high and low stimulus levels in the speech reception in noise tasks (CRM and DTT) observed in the current study is broadly consistent with the results of Johannesen et al. (2019), who did not find significant interactions between age and target speech level for the reception of words in steady-state or fluctuating noise maskers. Our findings are also consistent with those of Prendergast et al. (2019) for the CRM task.

Prendergast et al. (2019) found a differential effect of age at high and low stimulus levels in the DTT, with greater age-related threshold increases at the high stimulus level. Additionally, they found that greater levels of lifetime noise exposure were associated with greater threshold decreases at the high compared to the low stimulus level. We found no evidence for such effects in the current study, and the CIs for such effects in the current study were relatively narrow, especially for the differential age effect. There are several differences in the stimuli and procedures between the two studies (e.g. speech stimuli were lowpass filtered at 3 kHz and a band of pink noise was added above 3 kHz only in the current study), but none of them seems an obvious candidate to explain the different results. Because Prendergast et al. (2019) measured thresholds at the 25% correct point of the psychometric function, we repeated the DTT analyses using the 1/4 point of the psychometric function (i.e. ∼25% for a listener with a zero lapse rate) instead of the midpoint, but found the results qualitatively unchanged. Another difference between the two studies is that we included cognitive abilities covariates in the analyses; however we did not include interaction terms between cognitive abilities and stimulus level, therefore this difference cannot explain the discrepancies in the results of the two studies. Perhaps, an explanation for the differing results may lie in the populations of participants tested in the two studies. Besides differences in the age range of the participants included in the two studies (see Introduction), the overall levels of lifetime noise exposure were lower in the current study compared to the study of Prendergast et al. (2019). This may reflect a different recruitment strategy that favored acquiring participants with extreme ranges of noise exposure in the Prendergast et al. (2019) study in order to test for effects of noise-induced CS. Interestingly, qualitative analyses suggested that the largest age-related differences in Prendergast et al. (2019) occurred between older listeners, who had high levels of noise exposure, and a subset of young highly noise-exposed listeners, rather than with young listeners who had low levels of noise exposure. However, the interaction between age and noise exposure in a multiple regression model was not significant; additionally, musical experience, which may have been a lurking variable driving either the noise or age effects, did not appear to explain a significant amount of variance. Overall, the available evidence does not point clearly to differences in the participant populations as a reason for the differing results in the two studies. However, it remains possible that a lurking variable other than musical experience, and which correlated with either age or noise exposure, may have driven the differential effects of age and noise exposure found by Prendergast et al. (2019).

Although the major aim of the current study was the assessment of possible effects of age-related CS, it is notable that no credible differential effects of lifetime noise exposure between high and low stimulus levels were found on any of the tests. Although the results of this single study do not exclude the possibility of small or moderate effects of noise exposure on such tests, they add to the results of the majority of studies on the topic, that have failed to find effects of lifetime noise exposure on behavioral measures of auditory processing (see Bramhall, Beach, Epp, Le Prell, Lopez-Poveda, Plack, Schaette, Verhulst, Canlon, 2019, Le Prell, 2019, for reviews).

### 4.2 General effects

Although the main objective of the current study was to test for the presence of differential effects of stimulus level on age-related auditory deficits that could be signs of CS, it is also of interest to assess the associations between performance in the psychophysical and speech-reception tests and the predictor variables at each stimulus level. The following sections will discuss these associations in relation to previous findings in the literature.

#### 4.2.1 Amplitude modulation detection

The results of the current study indicate the presence of age-related increases in AM detection thresholds for most of the combinations of level and modulation frequencies tested, and trends in the same direction for the remaining conditions. Studies assessing effects of age on AM detection for listeners with audiometric thresholds within the normal range at the test frequencies have yielded mixed results, with some finding evidence of age-related AM detection declines (Füllgrabe, Moore, Stone, 2014, He, Mills, Ahlstrom, Dubno, 2008, Wallaert, Moore, Lorenzi, 2016), and others not (Grose, Buss, Elmore, 2019, Paraouty, Ewert, Wallaert, Lorenzi, 2016, Schoof, Rosen, 2014).

Several factors could account for the differing results across studies, including differences in the age range of the older participants tested, small sample sizes in some studies, and differences in the specific stimuli used (e.g. sinusoidal vs noise carriers; tones in quiet vs tones in noise). Moore and Vinay (2019) have suggested that differences across studies could be partly explained by the fact that OHC dysfunction in older listeners can partially offset age-related deficits (though older listeners had thresholds within the normal range in these studies, they typically had higher thresholds than young listeners, indicative of OHC dysfunction). Using the envelope regularity discrimination test, a test that is similar to AM detection but according to Moore et al. (2019) should be little affected by OHC dysfunction, Moore and Vinay (2019) found higher thresholds in a group of older listeners compared to young listeners, but their sample size was small. The results of our study do not provide strong evidence that raised audiometric thresholds can offset age-related AM detection deficits, but are consistent with this hypothesis, because there were trends for independent effects of audiometric thresholds on AM detection thresholds in several conditions of the AM detection task.

#### 4.2.2 Frequency/F0 discrimination

In the current study, we did not find evidence that age *per se* affects pure tone frequency discrimination. On the other hand we found evidence that audiometric thresholds are associated with pure tone frequency discrimination independently of age not only at low, but also at high stimulus levels. Overall these results are consistent with those of Marmel et al. (2013). Two other studies, however, reported age effects on frequency discrimination thresholds by comparing groups of younger and older listeners with thresholds within the normal range (Clinard, Tremblay, Krishnan, 2010, He, Dubno, Mills, 1998). One of these studies (He et al., 1998) matched quite closely audiometric thresholds between the two groups at some of the test frequencies, but had a very small sample size. For the other study (Clinard et al., 2010) no information on the audiometric thresholds is available beyond the fact that participants had thresholds <25 dB HL up to 8 kHz, so it is possible that residual audiometric differences could have accounted for the age effect.

We did not find evidence of age effects on F0 discrimination of unresolved complex tones in the current study, consistent with the results of Bianchi, Carney, Dau, Santurette, 2019. Although our results rule out the hypothesis that age *per se* may have large effects on F0 discrimination of unresolved complex tones, the CIs for the age effect were not very narrow, and are compatible with the possibility that small age effects exist but were not detected in the current study.

#### 4.2.3 Interaural phase difference detection

In the current study we found evidence of age-related declines in MOD IPD detection for a tone with a relatively high carrier frequency (2 kHz), but no evidence of age effects for tones with a low carrier frequency (600 Hz).

Several studies reported age-related deficits on IPD detection for low-frequency pure tones using either the TFS-LF test (see Füllgrabe and Moore, 2018, for a review and meta-analysis of these studies) or similar tests (Grose, Mamo, 2010, King, Hopkins, Plack, 2014). Overall, their results indicate that age and audiometric thresholds at the test frequency make independent contributions to performance in these tests. Potential explanations for the fact that independent effects of age were generally detected in these studies but not in the current one will be given below. However, it should be first pointed out that our results and those of these studies are not necessarily discrepant, because the CIs in the current study, while incompatible with large effects of age on low-frequency IPD detection are not incompatible with the possibility that small or moderate effects exist.

Several of the studies reviewed in Füllgrabe and Moore (2018), and also the study of King et al. (2014), included somewhat older participants (up to 90 years old) than those of the current study. Given that age-related deficits in the detection of a 180° IPD tend to occur first at high carrier frequencies, before progressing towards lower ones (Füllgrabe et al., 2018, Grose, Mamo, 2010, Ross, Fujioka, Tremblay, Picton, 2007), it is possible that the lower age range of our participant sample allowed us to detect age-related IPD deficits at high, but not at low frequencies.

Another important difference between the current and previous studies is that in the current study age effects were estimated while partialing out not only effects of audiometric thresholds, but also effects of cognitive abilities and musical experience. Several studies (Füllgrabe, Moore, Stone, 2014, Strelcyk, Zahorik, Shehorn, Patro, Derleth, 2019) have found associations between cognitive abilities and IPD detection, and although our results do not provide conclusive evidence for this association, the trends in the data are consistent with it. It is not clear whether the age effects found in previous studies would have survived if the effects of cognitive abilities had been partialed out.

We found effects of audiometric thresholds on IPD MOD detection at low stimulus levels, and a trend in the same direction for PT IPD detection at a low stimulus level. Overall these results are consistent with those of previous studies (Füllgrabe and Moore, 2018, King, Hopkins, Plack, 2014). However, these studies presented stimuli at equal sensation levels rather than at equal SPLs, as was done in the current study. Therefore it is difficult to directly compare their results with those of the current study *re* audiometric threshold effects, because in the current study, effects of audiometric thresholds would include the effect of reduced sensation level in addition to other psychophysiological changes associated with raised audiometric thresholds.

#### 4.2.4 Speech reception tasks

The results of the current study on the DTT do not provide evidence of age effects independent of audiometric threshold shifts. On the other hand, poor performance on this test at the lower stimulus level was associated with higher audiometric thresholds independently of age. These results are in line with other studies that have not found evidence that age *per se* affects speech reception in a steady-state noise background, and that the performance declines of older adults in these conditions can be largely accounted for by audiometric threshold shifts (Humes and Dubno, 2010).

Performance in the CRM test is limited mainly by informational masking (Brungart et al., 2006). Previous studies using the CRM test have generally found that age effects are minimal or absent when the target and maskers are colocated and are of the same gender, while older adults perform worse than younger adults in conditions in which target and maskers are spatially offset or are of different genders (Gallun, Diedesch, Kampel, Jakien, 2013, Humes, Kidd, Lentz, 2013, Humes, Lee, Coughlin, 2006, Marrone, Mason, Kidd, 2008, Rossi-Katz, Arehart, 2009). These findings suggest that release from informational masking on the basis of F0 or spatial cues is impaired in older listeners. However, it is unclear to what extent these differences between younger and older adults could be accounted for by audiometric threshold elevations, and one study failed to find effects of age on spatial release from masking independent of hearing loss (Jakien et al., 2017). Many of the studies cited above compared younger and older listeners with low-frequency audiometric thresholds within the normal range, or used other techniques to try to estimate age effects independently of audiometric threshold shifts, such as attempting to match audibility via spectral shaping, but many of these approaches could not completely exclude the possibility that residual audiometric threshold differences were contributing to the age effect. Additionally, some of these studies did not attempt to estimate age effects independent of age-related cognitive declines. Using a strict approach to test for the presence of age effects independent of effects of audiometric thresholds and cognitive abilities, the results of the current study do not provide evidence that aging *per se* affects performance in the CRM test either when the target and maskers are colocated (and could be of a different gender), or when they are spatially offset. On the other hand, our results confirm the finding that audiometric threshold shifts are associated, independently of age, with deficits in spatial release from masking (Marrone et al., 2008).

#### 4.2.5 Consonance perception

In the current study we found that consonance preference credibly decreased with increasing age at the high stimulus level, and a trend in the same direction was present at the low stimulus level. These effects, as well as the credible increase in consonance preference with years of musical experience that we found in the current study, are consistent with previous reports (Bones, Hopkins, Krishnan, Plack, 2014, Bones, Plack, 2015), and additionally show that these effects are independent of potential confounding effects of audiometric threshold elevations and cognitive abilities.

We did not find credible effects of audiometric thresholds on consonance preference. The results of a previous study (Tufts et al., 2005) suggest that audiometric threshold losses reduce the ability to perceive the contrast between consonant and dissonant intervals. However, the effect may have been driven by covariations between age and audiometric threshold losses, that were not taken into account in that study. On the other hand, Tufts et al. (2005) included participants with larger audiometric losses than those of the participants tested in the current study. It is possible that effects of audiometric threshold elevations on consonance perception become apparent only for larger losses, and this may explain why they were not detected in the current study.

#### 4.2.6 Role of cognitive factors

In the current study, credible independent effects of cognitive abilities on auditory processing were only found for AM detection and consonance preference. However, it is remarkable that for almost all tests there were trends for at least one of the two principal components of cognitive test scores to be positively associated with performance. While these results do not provide strong evidence (except for AM detection and consonance preference) for a role of cognitive factors on performance in psychophysical and speech reception tests, they are certainly compatible with it. This underlies the importance of controlling for potential effects of individual differences in cognitive abilities in observational studies assessing effects of age or noise exposure on psychophysical and speech reception performance.

#### 4.2.7 Self-reported hearing abilities

No credible effects of age or of any other independent variable were found on SSQ12 scores in the current study. This may seem somewhat surprising given that effects of age and/or audiometric threshold losses were found on several objective psychophysical and speech reception tests. These findings are consistent with those of Füllgrabe et al. (2014), who did not find significant differences in SSQ scores between a group of younger and older listeners with low-frequency thresholds within the normal range, despite finding differences between the two groups on several objective psychophysical and speech-reception tests. Banh et al. (2012), on the other hand, found significant differences in SSQ scores between a group of young and a group of older listeners with normal audiometric thresholds below 4 kHz. The differing results may be partly due to the fact that the participants tested by Banh et al. (2012) had somewhat higher audiometric thresholds than those tested in the current study, and those tested by Füllgrabe et al. (2014).

Overall the SSQ12 results of the current study suggest that for people with relatively well-preserved low-frequency audiometric thresholds, self-perceived hearing abilities are largely unaffected by age or by mild high-frequency audiometric threshold losses. It is unclear to what extent this is due to i) objective real-world hearing abilities being minimally affected by the age/audiometric deficits documented in the laboratory-based tests of this study, ii) insufficient sensitivity of the SSQ12 to detect mild self-perceived deficits, or iii) cognitive heuristics or socio-cultural factors that lead older people with hearing deficits to minimize them (see supplementary materials in Füllgrabe et al., 2014).

## 5 Conclusions

Overall the results of the current study on a cross-sectional sample of 102 participants are consistent with those of previous studies indicating the presence of age-related deficits independent of audiometric threshold shifts on several psychophysical auditory temporal processing tasks, but they do not provide evidence for age effects independent of audiometric threshold shifts on the reception of speech masked by either static noise, or interfering talkers.

The results of the current study do not provide evidence that performance on psychophysical temporal processing and speech-reception tasks follows a pattern consistent with age-related CS, which would predict greater age-related deficits at high compared to low stimulus levels, once the effects of audiometric threshold shifts or other potential confounders are accounted for. A caveat on this conclusion is that the predictions are based on a pathophysiological model of CS that has been developed mainly in rodents, and it is not clear to what extent it applies to humans. For example, as noted by Hickox et al. (2017), the association between the spontaneous rates of auditory-nerve fibers and their thresholds, which have been observed for a number of mammalian species, were not observed in a study on a non-human primate species (macaque; Joris et al., 2011). Therefore, it is not clear whether the pathophysiological model of age-related CS affecting mainly fibers with high thresholds applies to humans. Furthermore, as discussed in the Introduction, while there is substantial evidence suggesting that age-related CS in rodents is specific for L/M-SR fibers, currently this evidence is not conclusive. Overall, given the currently available evidence, it cannot be excluded that age-related CS in humans differs from noise-induced CS in rodents *re* L/M-SR specificity. If this is the case, some of the age-related changes observed in the current study could be attributed to CS that is not specific to L/M-SR fibers. However, as discussed in the Introduction, they could also reflect sensorineural deficits other than CS: if CS effects are not level specific, it becomes difficult to distinguish them from other sensorineural deficits on the basis of the measures employed in the current study.

## Declaration of Competing Interest

The authors declare that they have no conflict of interest.

## Appendix A Supplementary materials

Supplementary Data S1 Supplementary Raw Research Data. This is open data under the CC BY license http://creativecommons.org/licenses/by/4.0/ Supplementary Data S1

## Footnotes

1 Amplitude modulation increases the average power of the sound by a factor of $1+m^{2}/2$ (where m is the modulation index; Viemeister, 1979). Detection thresholds for intensity increments of pure tones in notched noise (similar to the condition of the current study in which the AM tone was flanked by low and high pass noise maskers) are around 1.4-1.5 dB for highly trained listeners (Moore and Raab, 1974, Schneider, Parker, 1987). Such intensity differences would be reached for AM depths exceeding ∼−1.2 dB; given that AM detection thresholds in the current study were generally much lower than -1.2 dB it can be excluded that they were determined by intensity cues. Because intensity-discrimination thresholds tend to worsen with increasing age (He et al., 1998), it is also unlikely that the very few participants with AM detection thresholds close to, or greater than 1.2 dB, who were generally older, used intensity cues to perform the task.

2 The noise was intended to be a speech shaped noise (SSN). However, due to a bug in version 1.5 of the software used to generate the SSN (https://it.mathworks.com/matlabcentral/fileexchange/37376-oscillator-and-signal-generator), its spectral shape was different from that of an SSN. Figure S1 in the supplementary materials shows the spectrum of an SSN and the spectrum of the noise used for the DTT task in the current study.

3 Bayesian credibility intervals have analogies with Frequentist confidence intervals, and Bayesian statements of credible differences have analogies with Frequentist statements of significant differences; however, there are also subtle but fundamental differences in the definition and interpretation of these concepts in the context of Bayesian and Frequentist analyses that cannot be succinctly described within the scope of this paper. Accessible expositions of these differences are provided by Kruschke, 2013, Kruschke, 2014, and Kruschke and Liddell (2018). McMillan and Cannon (2019) provide an introduction to Bayesian analysis methods in the context of auditory research.
