# Supplementary material for: Effects of age on psychophysical measures of auditory temporal processing and speech reception at low and high levels
Source: Hear Res. 2021 Feb;400:108117. doi: 10.1016/j.heares.2020.108117 (PMC7812372; doi:10.1016/j.heares.2020.108117)
Supplement: Supplementary Data S1 — Supplementary Raw Research Data. This is open data under the CC BY license http://creativecommons.org/licenses/by/4.0/ [file mmc1.pdf]

Supplementary materials for “Effects of age on psychophysical measures of auditory temporal processing and speech reception at low and high levels”

Contents

1 Supplementary figures referenced in the main manuscript **2**

2 Supplementary tables referenced in the main manuscript **14**

3 Supplementary methods **16**
3.1 UML settings 16

4 Psychometric function fits **17**

5 Statistical models and results **20**
5.1 Bayesian correlation model 20
5.2 Mixed effect multiple regression models 20

1 Supplementary figures referenced in the main manuscript

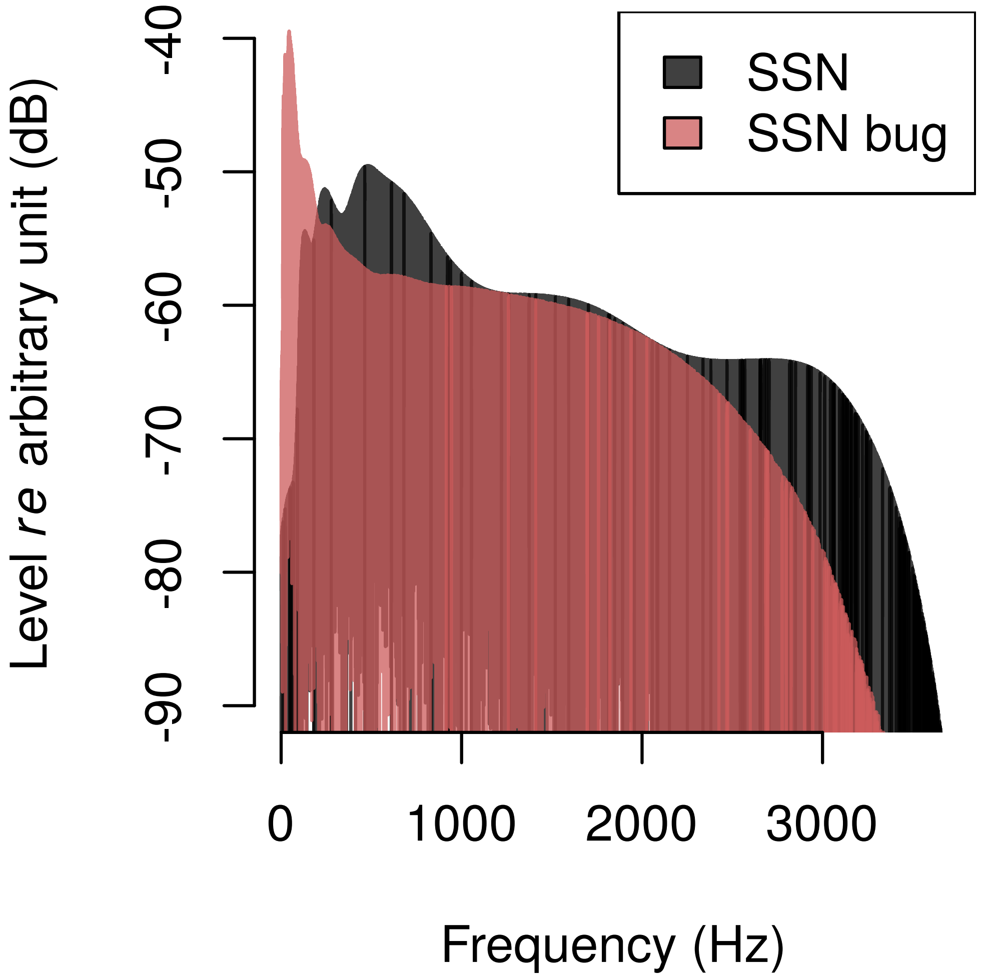

Figure S1: Spectrum of a speech-shaped noise (SSN; black line), and of the noise used for the digit triplets test in the current study (red line). Both noises are lowpass filtered at 3 kHz. Due to a bug in version 1.5 of the software (https://it.mathworks.com/matlabcentral/fileexchange/37376-oscillator-and-signal-generator) used to generate the SSN for the current study, its spectral shape had relatively higher energy below ∼200 Hz, and lower energy above ∼2.5 kHz compared to a SSN.

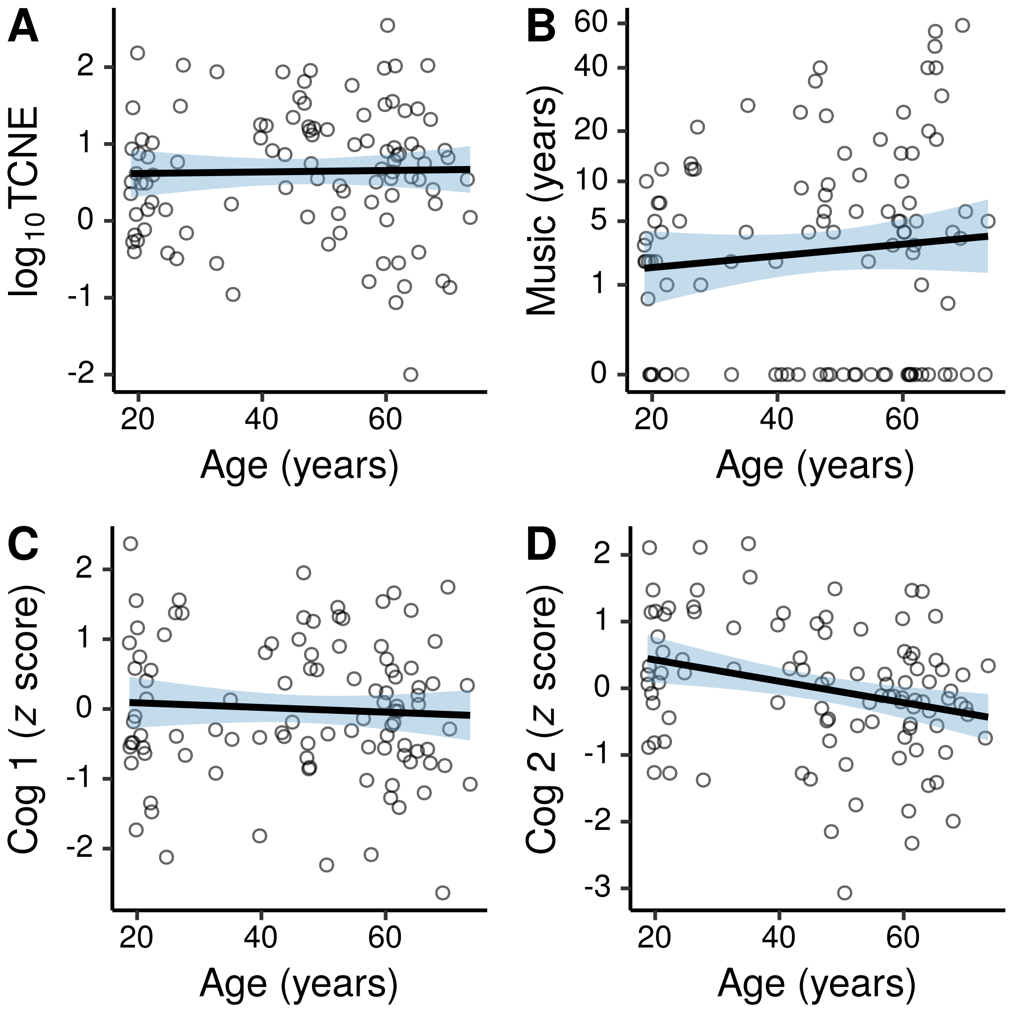

Figure S2: **A.** Total cumulative noise exposure as a function of age. A unit difference in the base 10 logarithmic y axis of the figure corresponds to a tenfold difference in noise exposure energy. **B.** Years of musical experience as a function of age. The y axis is shown on a cube root scale (years are displayed in their original unit). **C.** Standardized scores of the first principal component from the PCA of the cognitive test scores. **D.** Standardized scores of the second principal component from the PCA of the cognitive test scores.

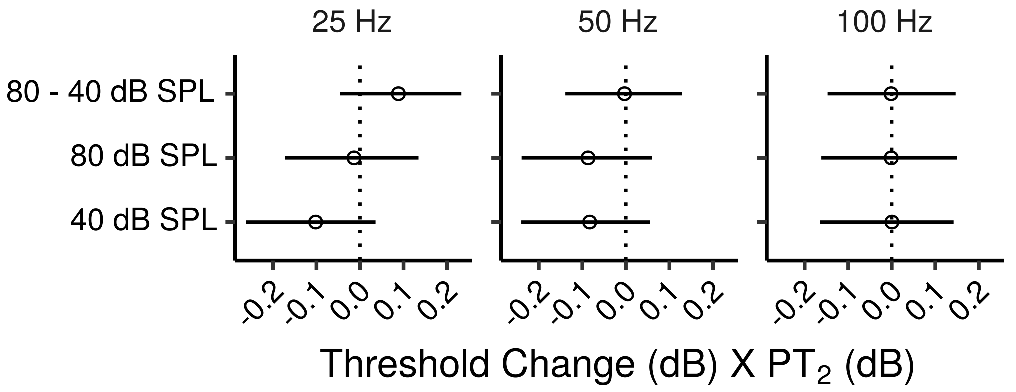

Figure S3: Posterior medians (circles) and 99% CIs for the effects of PT_2 on AM detection thresholds estimated by the Bayesian multiple regression model.

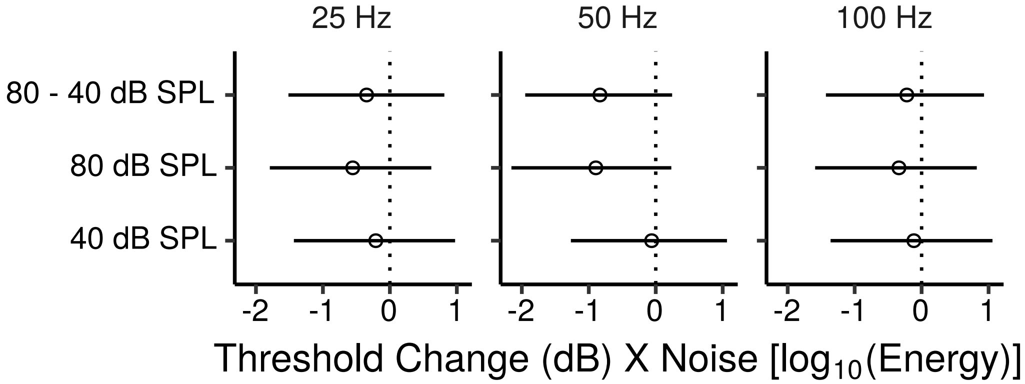

Figure S4: Posterior medians (circles) and 99% CIs for the effects of $\log_{10}$TCNE on AM detection thresholds estimated by the Bayesian multiple regression model.

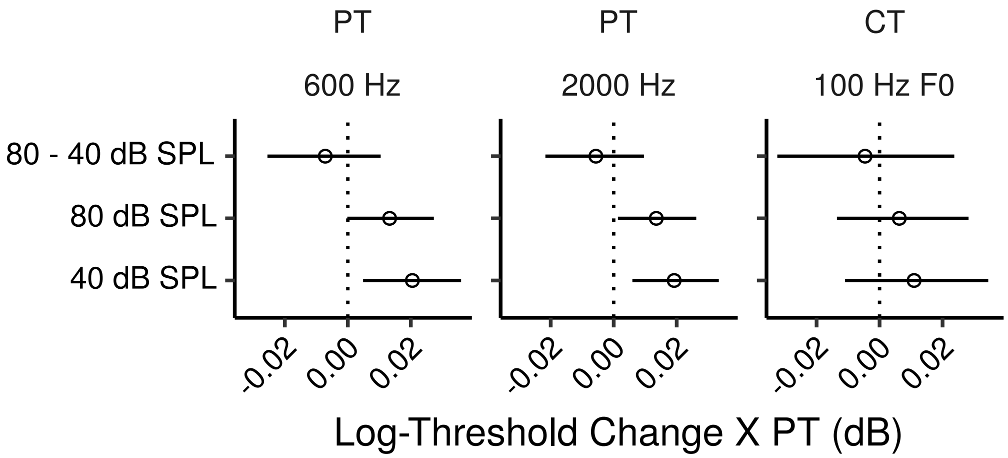

Figure S5: Posterior medians (circles) and 99% CIs for the effects of audiometric thresholds (at 0.5 kHz for the 0.6 kHz pure tone, and at 2 kHz for the other two stimuli) on pure tone (PT) frequency discrimination thresholds, and complex tone (CT) F0 discrimination thresholds estimated by the Bayesian multiple regression models.

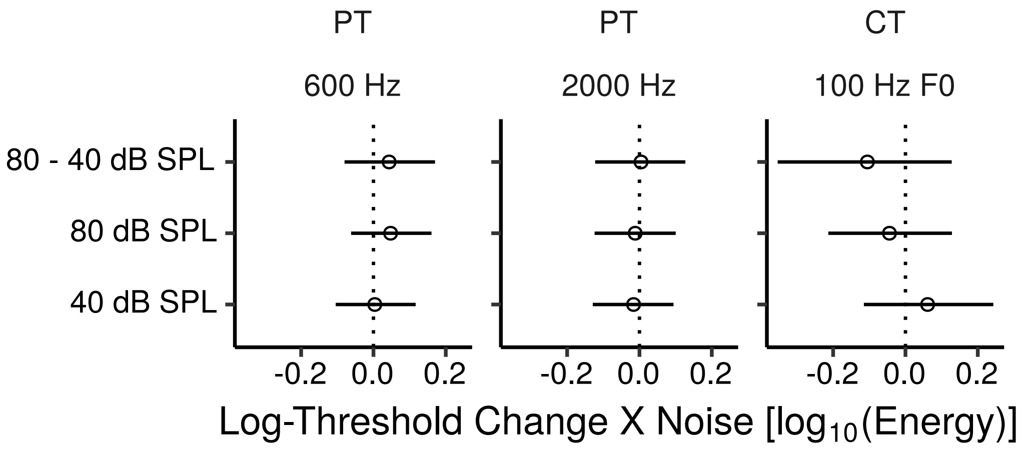

Figure S6: Posterior medians (circles) and 99% CIs for the effects of $\log_{10}$TCNE on pure tone (PT) frequency discrimination thresholds, and complex tone (CT) F0 discrimination thresholds estimated by the Bayesian multiple regression models.

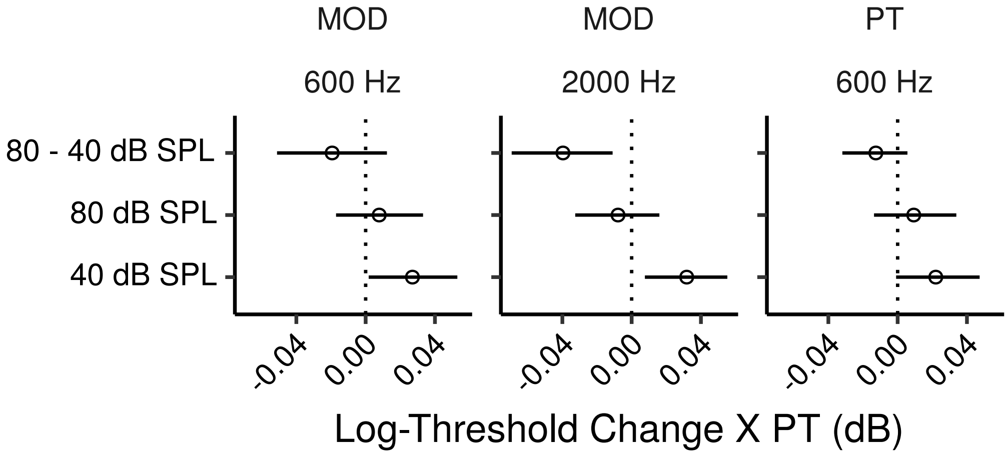

Figure S7: Posterior medians (circles) and 99% CIs for the effects of audiometric thresholds (at 2 kHz for the 2-kHz carrier, and at 0.5 kHz for the other two stimuli) on IPD detection thresholds estimated by the Bayesian multiple regression models. MOD refers to conditions in which the IPD was applied to the modulator of an AM tone, and PT to conditions in which the IPD was applied to a pure tone.

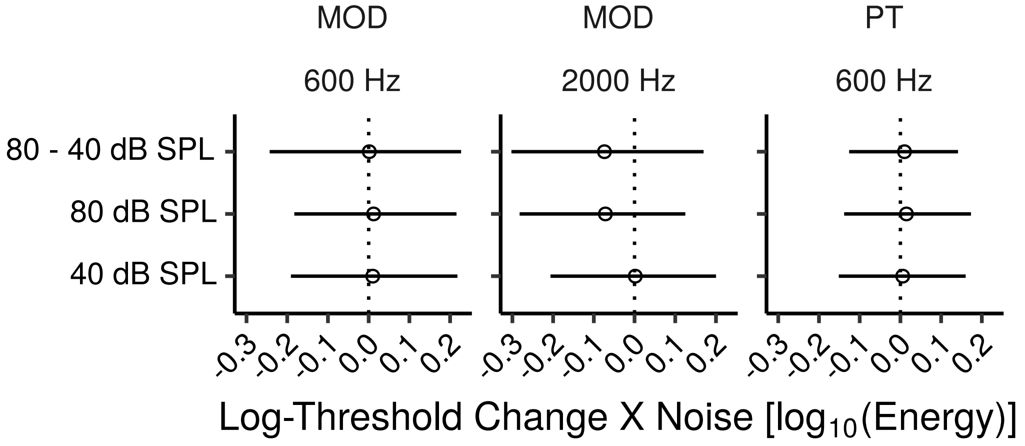

Figure S8: Posterior medians (circles) and 99% CIs for the effects of $\log_{10}$TCNE on IPD detection thresholds estimated by the Bayesian multiple regression models. MOD refers to conditions in which the IPD was applied to the modulator of an AM tone, and PT to conditions in which the IPD was applied to a pure tone.

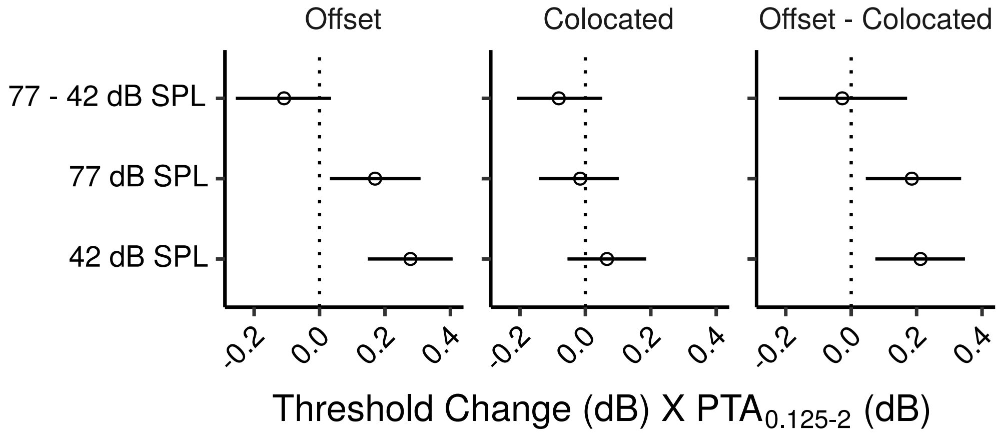

Figure S9: Posterior medians (circles) and 99% CIs for the effects of $PTA_{0.125-2}$ on CRM thresholds estimated by the Bayesian multiple regression model. The leftmost, and the central panel show effects for the conditions with maskers that were either offset, or colocated with the target, respectively. The rightmost panel shows the effect difference between these two conditions. Values above zero in this panel indicate that spatial release from masking decreases with increasing $PTA_{0.125-2}$.

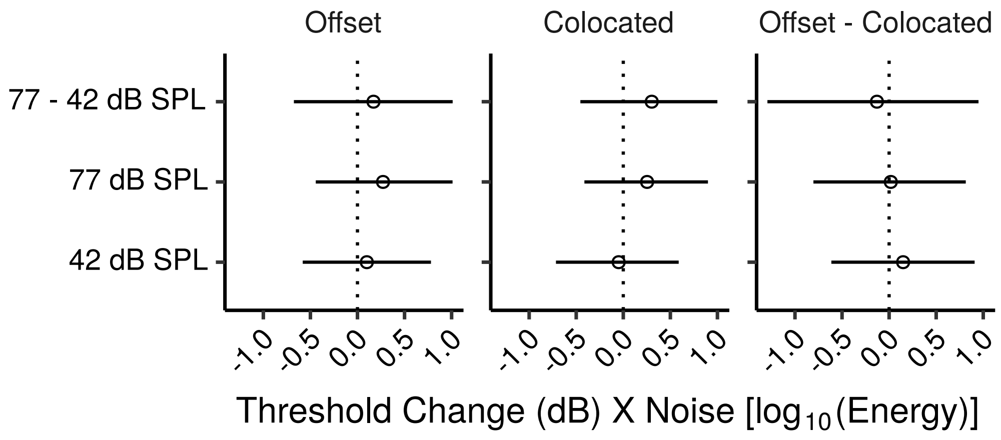

Figure S10: Posterior medians (circles) and 99% CIs for the effects of $\log_{10}$TCNE on CRM thresholds estimated by the Bayesian multiple regression model. The leftmost, and the central panel show effects for the conditions with maskers that were either offset, or colocated with the target, respectively. The rightmost panel shows the effect difference between these two conditions. Values above zero in this panel would indicate that spatial release from masking decreases with increasing $\log_{10}$TCNE.

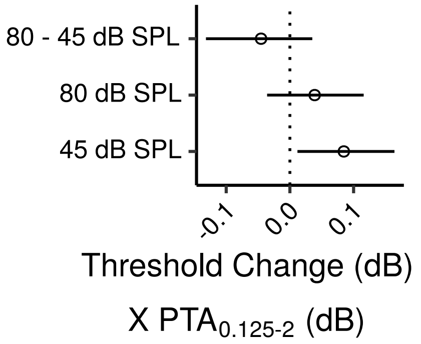

Figure S11: Posterior medians (circles) and 99% CIs for the effects of $PTA_{0.125-2}$ on DTT thresholds estimated by the Bayesian multiple regression model.

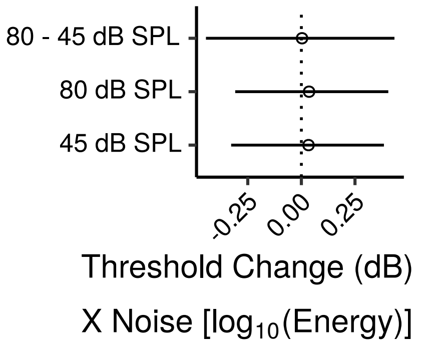

Figure S12: Posterior medians (circles) and 99% CIs for the effects of $\log_{10}$TCNE on DTT thresholds estimated by the Bayesian multiple regression model.

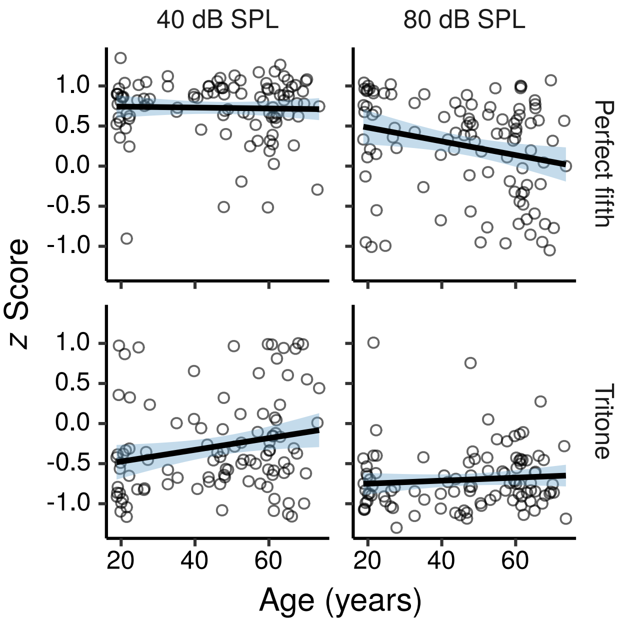

Figure S13: Standardized ratings for musical intervals by age. Each panel shows a least squares line fit of standardized ratings by age with 95% confidence intervals as a visual aid. The slope for the effect of age estimated by the Bayesian multiple regression model is not the same as that shown in the figure.

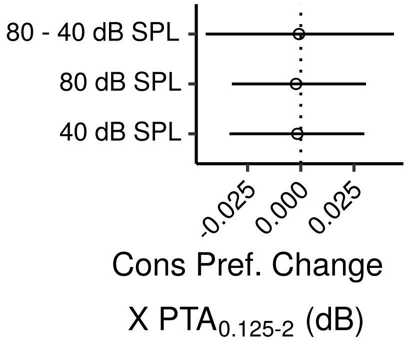

Figure S14: Posterior medians (circles) and 99% CIs for the effects of $PTA_{0.125-2}$ on consonance preference scores estimated by the Bayesian multiple regression model.

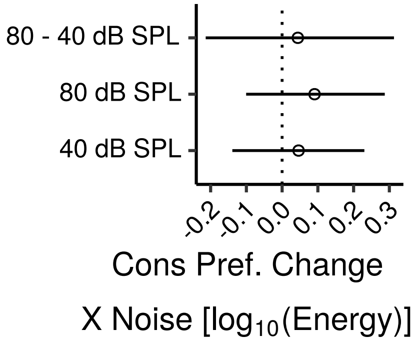

Figure S15: Posterior medians (circles) and 99% CIs for the effects of $\log_{10}$TCNE on consonance preference scores estimated by the Bayesian multiple regression model.

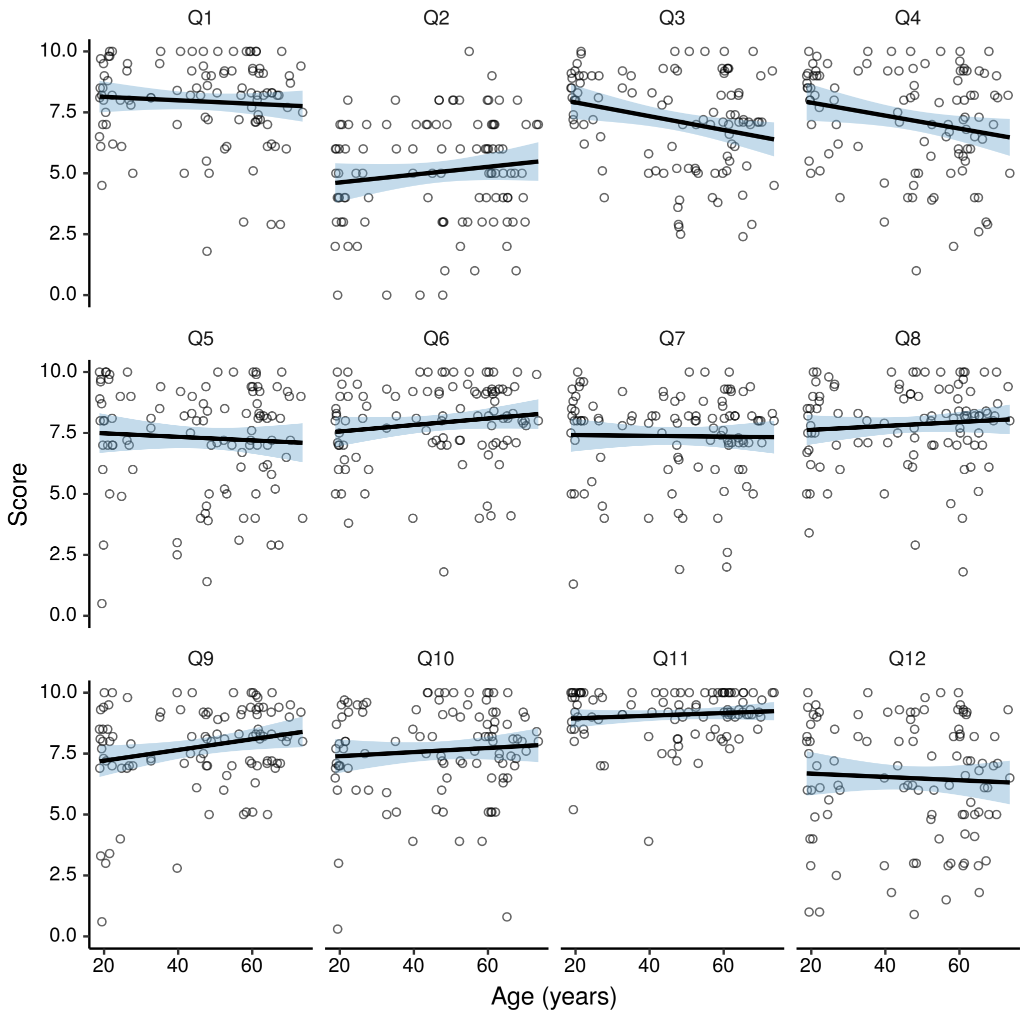

Figure S16: Scores for each question of the SSQ12 by age. Each panel shows a least squares line fit of SSQ12 score by age with 95% confidence intervals as a visual aid.

2 Supplementary tables referenced in the main manuscript

	Eigenvalue	% Variance	Cumulative % variance
PC1	2.00	50.00	50.00
PC2	0.96	23.97	73.97
PC3	0.62	15.41	89.37
PC4	0.43	10.63	100.00

Table S1: Eigenvalues, percentage of variance explained, and cumulative percentage of variance explained for the PCs extracted from the PCA of cognitive tests scores.

Task	PC1	PC2	PC3	PC4
Dig. span forw.	0.69	-0.59	0.11	0.40
Dig. span backw.	0.79	-0.31	-0.27	-0.44
Raven's prog. matr.	0.69	0.40	0.59	-0.11
Read. span	0.64	0.60	-0.43	0.23

Table S2: Loadings (correlations) between PCs and variables of the PCA of cognitive tests scores.

	Cog 1	Cog 2	MUS
**AMD**	-1.178 (-2.111 – -0.184)	0.115 (-0.710 – 0.983)	-0.157 (-0.879 – 0.516)
**PT FD**	-0.069 (-0.152 – 0.006)	-0.054 (-0.137 – 0.020)	-0.031 (-0.101 – 0.029)
**CT F0D**	-0.013 (-0.122 – 0.092)	-0.106 (-0.244 – 0.012)	-0.001 (-0.097 – 0.096)
**MOD IPD**	-0.076 (-0.210 – 0.048)	-0.053 (-0.195 – 0.070)	-0.025 (-0.137 – 0.082)
**PT IPD**	-0.081 (-0.225 – 0.040)	-0.101 (-0.253 – 0.035)	-0.033 (-0.152 – 0.069)
**CRM Col.**	-0.272 (-0.767 – 0.180)	-0.217 (-0.686 – 0.294)	-0.098 (-0.521 – 0.296)
**CRM Off.**	-0.278 (-0.799 – 0.210)	-0.193 (-0.746 – 0.319)	-0.404 (-0.843 – 0.030)
**CRM Off. - Col.**	-0.009 (-0.486 – 0.463)	0.023 (-0.486 – 0.545)	-0.306 (-0.737 – 0.120)
**DTT**	-0.210 (-0.502 – 0.061)	-0.122 (-0.414 – 0.119)	0.147 (-0.070 – 0.407)
**Cons. Pref.**	0.148 (0.031 – 0.269)	0.061 (-0.059 – 0.182)	0.150 (0.046 – 0.256)
**SSQ12**	0.056 (-0.187 – 0.330)	0.039 (-0.194 – 0.326)	-0.020 (-0.258 – 0.188)

Table S3: Posterior medians and 99% CIs (in brackets) for the effects of Cog1, Cog2, and MUS estimated by the Bayesian multiple regression models on AM detection thresholds (AMD), pure tone frequency discrimination thresholds (PT FD), complex tone F0 discrimination thresholds (CT F0D), IPD MOD and IPD PT detection thresholds, CRM thresholds in the colocated (Col.), and offset (Off.) conditions, and for the difference between offset and colocated conditions (Off. - Col.), DTT thresholds, consonance preference (Cons. Pref.), and SSQ12 scores.

3 Supplementary methods

3.1 UML settings

The form of the psychometric function assumed for all tasks was Logistic:

$$\psi(x;\alpha,\beta,\gamma,\lambda) = \gamma + (1-\gamma-\lambda)\left(\frac{1}{1+e^{\beta(\alpha-x)}}\right) \tag{1}$$

The parameter space for the UML procedure consisted of a three-dimensional grid defining the possible values of the midpoint (α), the parameter controlling the slope (β), the lapse rate (λ), and their prior probabilities. It should be noted that because the psychometric functions were re-fit after data collection (as suggested in Shen and Richards, 2012; Shen et al., 2015), the priors used for the UML procedures did not directly affect the final threshold estimates, but could affect the adaptive placement of the stimuli and the efficiency of the procedure. Data collection occurred over two blocks of trials for each task and condition. To maximize the efficiency of the adaptive procedure, the posterior distribution of the parameters at the end of the first block was saved, and was used as the prior for the second block of trials.

Besides the limits on the space of the parameters of the psychometric function, lower and upper limits on the values that the stimuli could take were defined. These upper limits also served to define the λ sweetpoint for all the tasks, except for the frequency and F0 discrimination task. For these two tasks the λ sweetpoint was initially set at a "suggested" frequency/F0 difference that was deemed sufficiently high to result in asymptotic performance for most listeners (10% frequency difference for the frequency discrimination task, and 80% F0 difference for the F0 discrimination task). However, if the current estimate of the psychometric function indicated that the frequency/F0 difference needed to reach a close-to-asymptotic performance (proportion correct of 0.99-λ) was greater than the suggested λ sweetpoint, the λ sweetpoint changed to the estimated frequency/F0 difference needed to reach a close-to-asymptotic performance. In this way most listeners would not be presented with very large frequency/F0 excursions across trials, but frequency/F0 differences larger than the suggested λ sweetpoint could be used if the current estimate of the psychometric function suggested that these were needed to more accurately estimate λ.

The parameter space for the slope and the lapse rate parameters, as well as their priors were the same for all tasks. The array of values for β ranged

from 0.1 to 10 with a logarithmic spacing (step factor of 1.1). The prior for β was uniform in this log space. The array of values for λ ranged linearly from 0.001 to 0.3 with a 0.01 step. A beta prior with a mean of 0.08 and a standard deviation of 0.065 was used for λ.

The parameter space for the midpoint in each task is detailed in Table S4. The midpoint (and the stimulus space) was defined in terms of the modulation index m for the AM detection task, in terms of the frequency or F0 difference (ΔF or $\Delta F0$), measured in percent, for the frequency/F0 discrimination tasks, in terms of the IPD, measured in degrees, for the IPD detection task, and in terms of the SNR of the target speech, measured in dB, for the CRM and DTT tasks.

Task	Par.	α	Step	Spacing	x
AM det.	m	0.01–1	1.05	log	0.001–1
Freq. discr.	ΔF (%)	0.005–50	1.05	log	0.0005–99
F0 discr.	$\Delta F0$ (%)	0.5–99	1.05	log	0.05–99
IPD det.	IPD (°)	1–180	1.05	log	0.1–180
CRM	SNR (dB)	-33-12	1	linear	-33-12
DTT	SNR (dB)	-40-10	1	linear	-40-10

Table S4: Table listing the settings for the parameter space of the midpoint (α), and of the stimuli for each task. The second column indicates the parameter that was varied in the task. The third column indicates the lower and upper limits of the midpoint array. The fourth column indicates the step size; for parameters with linear spacing (see fifth column) the step was additive, while for parameters with log spacing the step was a multiplicative factor. The prior was always uniform on the (log or linear) parameter space of the midpoint. The sixth column indicates the lower and upper limits of the parameter space for the stimuli (e.g. for the AM detection task, the lowest and highest values that m could take in any trial).

4 Psychometric function fits

A Logistic function (Eq. 1) was used for all fits. Fits were obtained via MCMC methods (Kuss et al., 2005) using JAGS (Plummer, 2003) and R (R Core Team, 2020). For the λ parameter, a gamma prior with a mode of 0.01 and a standard deviation of 0.08 was used for all tasks. The priors for the α and β parameters

were informed by preliminary fits obtained via maximum likelihood (Wichmann and Hill, 2001). For the β parameters gamma priors with the mode set at the geometric (across-participants) mean of the maximum likelihood estimate, and a standard deviation of 3 were used for all tasks. For the α parameters, normal (on a linear or log scale depending on the task; see Table S5) priors centered at the (across-participants) mean of the maximum likelihood estimate, and with a standard deviation indicated in Table S5 were used. Example priors for one condition of the pure tone frequency discrimination task are shown in Fig. S17.

Task	Par.	α s.d.	γ	Scaling
AM det.	m	4	1/3	log
Freq. discr.	ΔF (%)	4	1/2	log
F0 discr.	$\Delta F0$ (%)	4	1/2	log
IPD det.	IPD (°)	4	1/2	log
CRM	SNR (dB)	12	1/16	linear
DTT	SNR (dB)	12	1/336	linear

Table S5: Table listing the settings for the psychometric function fits for each task. The second column indicates the dependent variable. The third column indicates the standard deviation of the normal prior on α; for variables with logarithmic scaling, this value represents the geometric standard deviation. The fourth column indicates the value of the γ (guess rate) parameter of the psychometric function, that was fixed at the reciprocal of the number of response alternatives. The last column indicates whether the dependent variable was log-transformed before the fit.

Goodness of fit was assessed qualitatively, via visual posterior predictive checks (Kruschke, 2013), as well as via the Monte Carlo simulations described by Wichmann and Hill (2001). For the latter, 10,000 simulated datasets were generated using the parameter estimates of the fitted psychometric function. The deviance of each simulated dataset relative to the fitted function was calculated in order to derive the deviance distribution. The deviance of the observed data relative to the fitted function was then compared to the deviance distribution. Deviance values above the 95th percentile of the deviance distribution may be indicative of poor fits. Only for 10 out of the total of 2448 fits across tasks had observed deviances above the 95th percentile of the deviance distribution. This indicates that in the vast majority of cases the quantitative goodness of fit check did not detect issues with the fits. Visual inspection of the few poor

fits indicated that these were either due to psychometric functions with non-monotonicities, or to cases in which performance was very poor even for the highest differences between the standard and comparison stimuli (Δs). The former may be due to confusion or to lapses occurring more frequently at higher stimulus levels or in one of the two blocks of trials. The qualitative posterior predictive checks confirmed that the majority of fits were good. These checks also revealed a number of cases, occurring mostly for MOD IPD detection at 2 kHz, and in the AM detection task, in which performance was poor even at the highest Δs. Because of this, in some cases the adaptive procedure could not explore a wide range of performance points on the psychometric function, and only a few points at high Δs were available. Although in these cases the fits were usually good, in the sense that the fitted function passed close to available data points, the CIs around the parameter estimates were large. This reflects the fact that these functions could be relatively well fit by a large range of combinations of values of the midpoint, slope, and lapse rate. For example, poor performance at the highest Δs in some cases could be well accounted for by either a very high lapse rate or a very shallow slope; the resulting trade-off between these parameter estimates results in large uncertainty in the value of these parameters. Such estimates will necessarily be more noisy. However, because the same procedures for fitting the functions were used for all participants, the estimates, even if noisy in some cases, are not biased with respect to age or to the other variables of interest in the study.

For some cases in which performance at the highest Δs was poor the estimated threshold fell above the possible stimulus range. This is a normal consequence of the fact that performance at the highest possible stimulus level was still below threshold. Extrapolations of thresholds beyond the stimulus range have been performed in previous studies (Hopkins and Moore, 2010; King et al., 2014), relying on an assumed relation between the proportion of correct response (Pc) and Δ, that is considered valid also beyond the possible stimulus range. The approach employed in the current study, that allows threshold estimates beyond the stimulus range, is in a way similar to that of these previous studies, but relies on less assumptions. In particular, in the current study i) the form of the relation between Δ and Pc is estimated from the data (or, when the data of a given participant do not sufficiently constrain the estimate, is influenced by the priors, which being based on average across-participant estimates reflect the overall across-participant trends) rather than being based on further assumptions, and ii) performance is in part explained by the estimated lapse rate, which was always assumed to be zero in previous studies extrapolating

thresholds beyond the stimulus range.

5 Statistical models and results

5.1 Bayesian correlation model

The Bayesian model to estimate the correlations among predictor variables was based on the model of Lee and Wagenmakers (2014, chap. 5) but used vague uniform priors for estimating the standard deviations of the variables instead of inverse-square-root-gamma priors.

5.2 Mixed effect multiple regression models

Tables S7, S8, S9, S10, S11, S12, S13, S14, and S15 list all the terms included in each statistical model (excluding the random effect of participant). The first column indicates the variable to which each coefficient refers (abbreviated as previously defined in the main text of the manuscript or as indicated for each model in Table S6; this table also indicates the dummy codes used to encode categorical variables through an unweighted effect coding scheme). The second column indicates the type of variable (continuous, categorical, or interaction). The third column indicates (for all the terms except the intercept) the scale parameter of the 1-degree-of-freedom t distribution used as a prior for the standardized slope coefficient; for the intercept term this column indicates the standard deviation of the zero-centered normal distribution used as a prior for the intercept. The fourth column indicates the same quantity as the third column, but in unstandardized mean-centered units. The fifth column indicates the median of the posterior distribution in unstandardized mean-centered units. The sixth column indicates the 99% CI for the coefficients in unstandardized mean-centered units.

Because Tables S7–S15 list all the terms included in each model the regression equations can be derived from these tables. Full equations for the regression models are not given here because they would be very long, but an example equation for a model containing only a term for age, one for stimulus level (a categorical predictor encoded as a dummy variable), their interaction, and a random effect of subject is given below:

$$y_{[i,s]} = \beta_0 + \beta_A A_{[s]} + \beta_L L_{[i]} + \beta_{LxA} L_{[i]} A_{[s]} + r_{[s]} + \varepsilon_{[i,s]} \tag{2}$$

where $y_{[i,s]}$ is the score for the sth subject at the ith stimulus level, $A_{[s]}$ is the age of the sth subject, and $L_{[i]}$ is the dummy code for the ith stimulus level (-1=low lev., 1=high lev.); β_0 is the intercept, β_A, β_L, and β_{LxA} are the regression coefficients for the effects of age, stimulus level, and their interaction, respectively; $r_{[s]}$ is the random effect of the sth subject, and $\varepsilon_{[i,s]}$ is the residual error term.

To give a sense of the prior distribution, Figure S18 plots t distributions with the same mean and degrees of freedom as the priors used in the current study for several values of the scale parameter. In each case the prior probability is highest for values around zero; while it is sharply centered around zero for small scale values, it becomes more diffuse as the scale value increases. Even when the scale value is relatively small, due to its heavy tails the t distributions can accommodate coefficients much larger than zero if the likelihood provides clear evidence for this. For a more in depth overview of t priors see Kruschke (2014).

**Abbreviation**	**Variable**	**Dummy codes**
A	Age decade	
$\sqrt[3]{\text{Y}}$	Cube root music years	
L	Level	-1=low lev., 1=high lev.
MF1	Mod. freq. 1	-1=100 Hz, 1=25 Hz 0=50 Hz
MF2	Mod. freq. 2	-1=100 Hz, 1=50 Hz 0=25 Hz
F	Frequency / Carrier frequency	-1=low freq., 1=high freq.
O	Offset	-1=colocated, 1=offset
I	Interval	-1=tritone, 1=perf. fifth

Table S6: Abbreviated variable names for the statistical models. The third column lists the dummy codes used for each categorical variable.

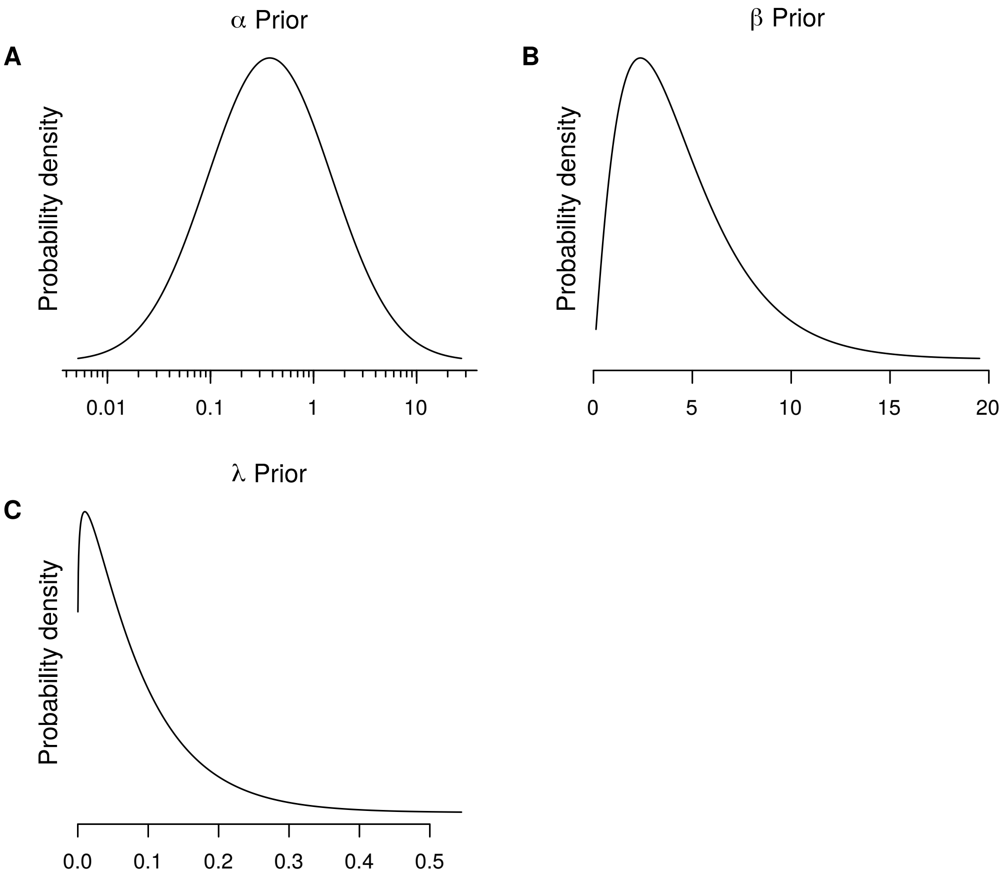

Figure S17: Priors for **A.** the α, **B.** β, and **C.** λ parameters of the Logistic psychometric function for the 0.6 kHz pure tone frequency discrimination task at a level of 80 dB SPL. The priors for λ were the same for all tasks and conditions. Priors for β had a the same standard deviation across tasks/conditions, but their mode was centered at the average value of the parameter for the task/condition obtained via preliminary maximum likelihood fits. Priors for α were similarly centered at the average value of the parameter for the task/condition obtained via preliminary maximum likelihood fits.

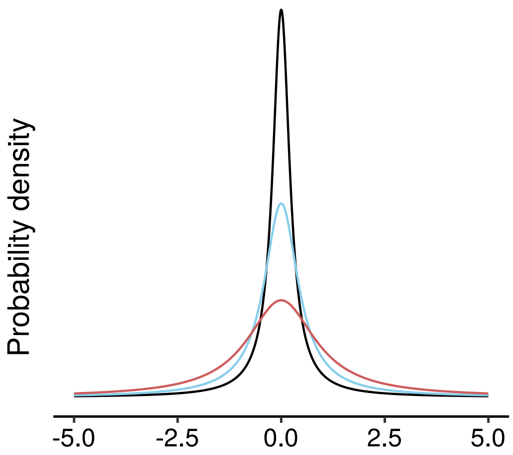

Figure S18: Density functions of t distributions with 1 degree of freedom centered at zero, and with scale parameter set to 0.25 (black), 0.5 (light blue), or 1 (red).

Table S7: Model terms and coefficients for the AM detection model.

**Coefficient**	**Type**	**Prior scale** z	**Prior scale**	**Posterior Median**	**99% CI**
Intercept		2.000	10.673	-12.073	-12.368– -11.787
A	Cont.	0.100	0.302	0.704	0.123–1.314
PT_2	Cont.	0.100	0.072	-0.047	-0.183–0.061
$\log_{10}$TCNE	Cont.	0.100	0.636	-0.357	-1.4–0.5
Cog1	Cont.	0.100	0.534	-1.178	-2.111– -0.184
Cog2	Cont.	0.100	0.534	0.115	-0.71–0.983
$\sqrt[3]{Y}$	Cont.	0.100	0.470	-0.157	-0.879–0.516
L	Cat.	10.000	53.365	2.340	2.051–2.625
MF1	Cat.	10.000	53.365	0.655	0.25–1.044
MF2	Cat.	10.000	53.365	-0.169	-0.554–0.249
LxMF1	Int.	0.100	0.534	-0.019	-0.403–0.355
LxMF2	Int.	0.100	0.534	0.021	-0.335–0.402
LxA	Int.	0.100	0.302	0.192	0.023–0.374
MF1xA	Int.	0.100	0.302	0.089	-0.138–0.323
MF2xA	Int.	0.100	0.302	-0.005	-0.223–0.224
LxMF1xA	Int.	0.100	0.302	-0.132	-0.365–0.108
LxMF2xA	Int.	0.100	0.302	0.136	-0.084–0.367
Lx$\log_{10}$TCNE	Int.	0.100	0.636	-0.233	-0.578–0.093
MF1x$\log_{10}$TCNE	Int.	0.100	0.636	-0.019	-0.481–0.449
MF2x$\log_{10}$TCNE	Int.	0.100	0.636	-0.115	-0.56–0.332
LxMF1x$\log_{10}$TCNE	Int.	0.100	0.636	0.061	-0.4–0.529
LxMF2x$\log_{10}$TCNE	Int.	0.100	0.636	-0.181	-0.64–0.262
LxPT_2	Int.	0.100	0.072	0.014	-0.026–0.054
MF1xPT_2	Int.	0.100	0.072	-0.010	-0.066–0.045
MF2xPT_2	Int.	0.100	0.072	-0.037	-0.092–0.017
LxMF1xPT_2	Int.	0.100	0.072	0.030	-0.027–0.087
LxMF2xPT_2	Int.	0.100	0.072	-0.015	-0.071–0.038

Table S8: Model terms and coefficients for the frequency discrimination model. The *PT* variable took the value of the pure tone audiometric threshold at 0.5 kHz for the 0.6-kHz pure tones, and at 2 kHz for the 2-kHz pure tones.

**Coefficient**	**Type**	**Prior scale** z	**Prior scale**	**Posterior Median**	**99% CI**
Intercept		2.000	0.964	-1.139	-1.177– -1.099
A	Cont.	0.100	0.027	0.007	-0.032–0.052
PT	Cont.	0.100	0.007	0.017	0.007–0.025
$\log_{10}$TCNE	Cont.	0.100	0.057	0.005	-0.076–0.088
Cog1	Cont.	0.100	0.048	-0.069	-0.152–0.006
Cog2	Cont.	0.100	0.048	-0.054	-0.137–0.02
$\sqrt[3]{Y}$	Cont.	0.100	0.042	-0.031	-0.101–0.029
L	Cat.	10.000	4.820	0.074	0.033–0.113
F	Cat.	10.000	4.820	0.014	-0.025–0.053
LxF	Int.	0.100	0.048	-0.013	-0.051–0.024
LxA	Int.	0.100	0.027	-0.009	-0.031–0.014
LxPT	Int.	0.100	0.007	-0.003	-0.009–0.003
Lx$\log_{10}$TCNE	Int.	0.100	0.057	0.012	-0.034–0.056
FxA	Int.	0.100	0.027	-0.006	-0.029–0.017
FxPT	Int.	0.100	0.007	-0.000	-0.007–0.006
Fx$\log_{10}$TCNE	Int.	0.100	0.057	-0.020	-0.066–0.025
LxFxA	Int.	0.100	0.027	-0.001	-0.024–0.021
LxFxPT	Int.	0.100	0.007	0.000	-0.005–0.006
LxFx$\log_{10}$TCNE	Int.	0.100	0.057	-0.010	-0.055–0.034

Table S9: Model terms and coefficients for the F0 discrimination model.

**Coefficient**	**Type**	**Prior scale** z	**Prior scale**	**Posterior Median**	**99% CI**
Intercept		2.000	1.671	1.353	1.247–1.463
A	Cont.	0.100	0.047	0.028	-0.039–0.109
PT_2	Cont.	0.100	0.011	0.009	-0.007–0.027
$\log_{10}$TCNE	Cont.	0.100	0.100	0.008	-0.119–0.137
Cog1	Cont.	0.100	0.084	-0.013	-0.122–0.092

Cog2	Cont.	0.100	0.084	-0.106	-0.244–0.012
$\sqrt[3]{Y}$	Cont.	0.100	0.074	-0.001	-0.097–0.096
L	Cat.	10.000	8.354	0.500	0.393–0.606
LxA	Int.	0.100	0.047	0.015	-0.045–0.077
LxPT$_2$	Int.	0.100	0.011	-0.002	-0.016–0.012
Lxlog$_{10}$TCNE	Int.	0.100	0.100	-0.053	-0.177–0.064

Table S10: Model terms and coefficients for the IPD MOD model. The *PT* variable took the value of the pure tone audiometric threshold at 0.5 kHz for the 0.6-kHz carrier, and at 2 kHz for the 2-kHz carrier.

**Coefficient**	**Type**	**Prior scale z**	**Prior scale**	**Posterior Median**	**99% CI**
Intercept		2.000	2.201	4.003	3.931–4.075
A	Cont.	0.100	0.062	0.076	-0.008–0.157
PT	Cont.	0.100	0.016	0.015	-0.001–0.03
log$_{10}$TCNE	Cont.	0.100	0.131	-0.012	-0.157–0.134
Cog1	Cont.	0.100	0.110	-0.076	-0.21–0.048
Cog2	Cont.	0.100	0.110	-0.053	-0.195–0.07
$\sqrt[3]{Y}$	Cont.	0.100	0.097	-0.025	-0.137–0.082
L	Cat.	10.000	11.004	-0.313	-0.382– -0.24
F	Cat.	10.000	11.004	0.743	0.671–0.815
LxF	Int.	0.100	0.110	-0.055	-0.127–0.015
LxA	Int.	0.100	0.062	0.028	-0.013–0.07
LxPT	Int.	0.100	0.016	-0.015	-0.026– -0.004
Lxlog$_{10}$TCNE	Int.	0.100	0.131	-0.018	-0.101–0.066
FxA	Int.	0.100	0.062	0.054	0.009–0.098
FxPT	Int.	0.100	0.016	-0.003	-0.015–0.009
Fxlog$_{10}$TCNE	Int.	0.100	0.131	-0.023	-0.109–0.059
LxFxA	Int.	0.100	0.062	0.021	-0.019–0.064
LxFxPT	Int.	0.100	0.016	-0.005	-0.016–0.005
LxFxlog$_{10}$TCNE	Int.	0.100	0.131	-0.019	-0.105–0.063

Table S11: Model terms and coefficients for the IPD PT model.

**Coefficient**	**Type**	**Prior scale** z	**Prior scale**	**Posterior Median**	**99% CI**
Intercept		2.000	1.361	3.266	3.202–3.328
A	Cont.	0.100	0.039	0.036	-0.034–0.122
$PT_{0.5}$	Cont.	0.100	0.010	0.016	-0.005–0.039
$\log_{10}$TCNE	Cont.	0.100	0.081	0.010	-0.13–0.154
Cog1	Cont.	0.100	0.068	-0.081	-0.225–0.04
Cog2	Cont.	0.100	0.068	-0.101	-0.253–0.035
$\sqrt[3]{Y}$	Cont.	0.100	0.060	-0.033	-0.152–0.069
L	Cat.	10.000	6.804	-0.178	-0.236– -0.117
LxA	Int.	0.100	0.039	-0.003	-0.036–0.029
$LxPT_{0.5}$	Int.	0.100	0.010	-0.006	-0.016–0.003
Lx$\log_{10}$TCNE	Int.	0.100	0.081	0.005	-0.063–0.071

Table S12: Model terms and coefficients for the CRM model.

**Coefficient**	**Type**	**Prior scale** z	**Prior scale**	**Posterior Median**	**99% CI**
Intercept		2.000	9.292	-6.066	-6.306– -5.814
A	Cont.	0.100	0.263	0.016	-0.23–0.274
$PTA_{0.125-2}$	Cont.	0.100	0.092	0.124	0.027–0.216
$\log_{10}$TCNE	Cont.	0.100	0.554	0.144	-0.333–0.635
Cog1	Cont.	0.100	0.465	-0.275	-0.701–0.151
Cog2	Cont.	0.100	0.465	-0.205	-0.658–0.216
$\sqrt[3]{Y}$	Cont.	0.100	0.410	-0.251	-0.624–0.105
L	Cat.	10.000	46.462	-1.226	-1.474– -0.985
O	Cat.	10.000	46.462	-3.542	-3.78– -3.296
LxO	Int.	0.100	0.465	-0.858	-1.107– -0.623
LxA	Int.	0.100	0.263	0.010	-0.132–0.148
OxA	Int.	0.100	0.263	0.044	-0.108–0.196
LxOxA	Int.	0.100	0.263	-0.051	-0.193–0.093
$LxPTA_{0.125-2}$	Int.	0.100	0.092	-0.047	-0.097–0.001
$OxPTA_{0.125-2}$	Int.	0.100	0.092	0.099	0.047–0.151
$LxOxPTA_{0.125-2}$	Int.	0.100	0.092	-0.007	-0.055–0.043
Lx$\log_{10}$TCNE	Int.	0.100	0.554	0.118	-0.169–0.394
Ox$\log_{10}$TCNE	Int.	0.100	0.554	0.042	-0.239–0.323

$LxOxlog_{10}TCNE$	Int.	0.100	0.554	-0.032	-0.323–0.237
OxCog1	Int.	0.100	0.465	-0.004	-0.243–0.231
OxCog2	Int.	0.100	0.465	0.012	-0.243–0.272
$Ox\sqrt[3]{Y}$	Int.	0.100	0.410	-0.153	-0.368–0.06

Table S13: Model terms and coefficients for the DTT model.

**Coefficient**	**Type**	**Prior scale** z	**Prior scale**	**Posterior Median**	**99% CI**
Intercept		2.000	3.016	-8.877	-9.093– -8.666
A	Cont.	0.100	0.085	0.078	-0.065–0.262
$PTA_{0.125-2}$	Cont.	0.100	0.030	0.062	-0.001–0.124
$log_{10}TCNE$	Cont.	0.100	0.180	0.033	-0.256–0.319
Cog1	Cont.	0.100	0.151	-0.210	-0.502–0.061
Cog2	Cont.	0.100	0.151	-0.122	-0.414–0.119
$\sqrt[3]{Y}$	Cont.	0.100	0.133	0.147	-0.07–0.407
L	Cat.	10.000	15.082	0.339	0.123–0.546
LxA	Int.	0.100	0.085	0.014	-0.098–0.128
$LxPTA_{0.125-2}$	Int.	0.100	0.030	-0.023	-0.066–0.018
$Lxlog_{10}TCNE$	Int.	0.100	0.180	0.002	-0.223–0.217

Table S14: Model terms and coefficients for the musical intervals ratings model.

**Coefficient**	**Type**	**Prior scale** z	**Prior scale**	**Posterior Median**	**99% CI**
Intercept		2.000	1.472	0.003	-0.053–0.057
A	Cont.	0.100	0.042	0.001	-0.032–0.035
$PTA_{0.125-2}$	Cont.	0.100	0.015	-0.001	-0.012–0.01
$log_{10}TCNE$	Cont.	0.100	0.088	-0.008	-0.073–0.061
Cog1	Cont.	0.100	0.074	0.004	-0.051–0.059
Cog2	Cont.	0.100	0.074	0.005	-0.055–0.06
$\sqrt[3]{Y}$	Cont.	0.100	0.065	-0.001	-0.05–0.045
L	Cat.	10.000	7.358	-0.204	-0.262– -0.146
I	Cat.	10.000	7.358	0.543	0.476–0.607
LxI	Int.	0.100	0.074	-0.013	-0.066–0.039
LxA	Int.	0.100	0.042	-0.024	-0.058–0.009

IxA	Int.	0.100	0.042	-0.048	-0.084– -0.011
LxIxA	Int.	0.100	0.042	-0.012	-0.043–0.021
LxPTA$_{0.125-2}$	Int.	0.100	0.015	-0.011	-0.022–0.001
IxPTA$_{0.125-2}$	Int.	0.100	0.015	-0.001	-0.012–0.01
LxIxPTA$_{0.125-2}$	Int.	0.100	0.015	-0.000	-0.011–0.011
Lxlog$_{10}$TCNE	Int.	0.100	0.088	0.065	-0.004–0.139
Ixlog$_{10}$TCNE	Int.	0.100	0.088	0.034	-0.034–0.102
LxIxlog$_{10}$TCNE	Int.	0.100	0.088	0.011	-0.053–0.078
IxCog1	Int.	0.100	0.074	0.074	0.015–0.134
IxCog2	Int.	0.100	0.074	0.031	-0.029–0.091
Ix$\sqrt[3]{Y}$	Int.	0.100	0.065	0.075	0.023–0.128

Table S15: Model terms and coefficients for the SSQ model.

**Coefficient**	**Type**	**Prior scale** z	**Prior scale**	**Posterior Median**	**99% CI**
Intercept		2.000	2.439	7.435	7.11–7.745
A	Cont.	0.100	0.069	0.013	-0.153–0.223
PTA$_{0.125-2}$	Cont.	0.100	0.024	-0.014	-0.075–0.034
PTA$_{4-12}$	Cont.	0.100	0.007	-0.002	-0.024–0.016
log$_{10}$TCNE	Cont.	0.100	0.145	-0.047	-0.367–0.229
Cog1	Cont.	0.100	0.122	0.056	-0.187–0.33
Cog2	Cont.	0.100	0.122	0.039	-0.194–0.326
$\sqrt[3]{Y}$	Cont.	0.100	0.107	-0.020	-0.258–0.188

References

Y. Shen and V. M. Richards. A maximum-likelihood procedure for estimating psychometric functions: thresholds, slopes, and lapses of attention. *J. Acoust. Soc. Am.*, 132(2):957–67, 2012. doi: 10.1121/1.4733540.

Y. Shen, W. Dai, and V. M. Richards. A MATLAB toolbox for the efficient estimation of the psychometric function using the updated maximum-likelihood adaptive procedure. *Behav. Res. Methods*, 47(1):13–26, 2015. doi: 10.3758/s13428-014-0450-6.

M. Kuss, F. Jäkel, and F. A. Wichmann. Bayesian inference for psychometric functions. *J. Vis.*, 5(5):478–492, 2005. doi: 10.1167/5.5.8.

M. Plummer. JAGS: A program for analysis of Bayesian graphical models using Gibbs sampling. In Kurt Hornik, Friedrich Leisch, and Achim Zeileis, editors, *Proceedings of the 3rd International Workshop on Distributed Statistical Computing*, Vienna, Austria, 2003.

R Core Team. *R: A Language and Environment for Statistical Computing*. R Foundation for Statistical Computing, Vienna, Austria, 2020. URL https://www.R-project.org/.

F. A. Wichmann and N. J. Hill. The psychometric function: I. Fitting, sampling, and goodness of fit. *Percept. Psychophys.*, 63(8):1293–313, 2001. doi: 10.3758/bf03194544.

J. K. Kruschke. Posterior predictive checks can and should be Bayesian: Comment on Gelman and Shalizi, 'Philosophy and the practice of Bayesian statistics'. *Br. J. Math. Stat. Psychol.*, 66(1):45–56, 2013. doi: 10.1111/j.2044-8317.2012.02063.x.

K. Hopkins and B. C. J. Moore. Development of a fast method for measuring sensitivity to temporal fine structure information at low frequencies. *Int. J. Audiol.*, 49(12):940–946, 2010. doi: 10.3109/14992027.2010.512613.

A. King, K. Hopkins, and C. J. Plack. The effects of age and hearing loss on interaural phase difference discrimination. *J. Acoust. Soc. Am.*, 135(1):342–351, 2014. doi: 10.1121/1.4838995.

M. D. Lee and E.-J. Wagenmakers. *Bayesian cognitive modeling: A practical course*. Cambridge University Press, 2014. doi: 10.1017/CBO9781139087759.

J. K. Kruschke. *Doing Bayesian data analysis, a tutorial with R, JAGS, and Stan*. Elsevier, London, 2nd edition, 2014. ISBN 978-0-12-405916-0.
